# Biomolecular phenotyping and heterogeneity assessment of mesenchymal stromal cells using label-free Raman spectroscopy

**DOI:** 10.1038/s41598-021-81991-1

**Published:** 2021-02-23

**Authors:** R. A. Rocha, J. M. Fox, P. G. Genever, Y. Hancock

**Affiliations:** 1 Department of Physics, University of York, Heslington, York YO10 5DD UK; 2 Federal University of Technology-Paraná, Campus Dois Vizinhos, Paraná, 85660-000 Brazil; 3 Department of Biology, University of York, Heslington, York YO10 5DD UK; 4 York Biomedical Research Institute, University of York, Heslington, York YO10 5DD UK; 5 York Cross-disciplinary Centre for Systems Analysis, University of York, Heslington, York YO30 5GG UK; 6 School of Cancer and Pharmaceutical Sciences, Faculty of Life Sciences and Medicine, King’s College London, London, SE19RT UK

**Keywords:** Biophysics, Cell biology, Raman spectroscopy, Predictive markers, Mesenchymal stem cells

## Abstract

Easy, quantitative measures of biomolecular heterogeneity and high-stratified phenotyping are needed to identify and characterise complex disease processes at the single-cell level, as well as to predict cell fate. Here, we demonstrate how Raman spectroscopy can be used in the difficult-to-assess case of clonal, bone-derived mesenchymal stromal cells (MSCs) to identify MSC lines and group these according to biological function (e.g., differentiation capacity). Biomolecular stratification is achieved using high-precision measures obtained from representative statistical sampling that also enable quantified heterogeneity assessment. Application to primary MSCs and human dermal fibroblasts shows use of these measures as a label-free assay to classify cell sub-types within complex heterogeneous cell populations, thus demonstrating the potential for therapeutic translation, and broad application to the phenotypic characterisation of other cells.

## Introduction

The ability to accurately and quantitatively assess molecular-scale differences at the single-cell and cell-population levels is of immense importance in biomedical research. No two cells are the same, with cell-level variations typically found in normal cell-populations due to spontaneous genetic differences upon cell division, or from environmental factors, such as epigenetic modifications^1,2^. Even clonal populations consisting of cells derived from the same parent-cell have been found to exhibit cell-level differences^1–3^, with these also being important in the study of diseases that manifest at the single-cell level, such as cancer. Although the quantification and stratification of inter- and intra-cell heterogeneity are challenging, these remain key to understanding disease processes, including disease initiation, as well in the development of diagnostics and treatments^4,5^.

Cellular heterogeneity is typically assessed using microscopy and flow cytometry, which are not label-free^6^. DNA and RNA sequencing, and gene microarray technologies, have advanced cell assessment; however, these methods are data-intensive, involve detailed sample preparation via multi-step processes, are time-consuming and relatively costly^7^. Gawad et al.^8^ and Lo and Zhou^9^ provide detailed reviews of the state-of-the-art in single-cell genome sequencing, where the enormity of the challenge in determining and comparing full cell-level information is relayed. The relationship between the cell genotype and phenotype is also challenging to assess^10^. For example, it is difficult to ascertain whether all relevant gene-level information has been captured in the characterisation of cell-level disease. Changes to cellular expression, i.e., phenotypic plasticity, may also result from treatment-related genetic-scale changes^11^. From these examples alone, the enormity in developing tractable, quantitative assessment and phenotyping of cell-level and population-level heterogeneity, and the relationship of these factors to accurate assay development, is apparent.

To assist with these challenges, spectroscopic methods, such as Raman spectroscopy (RS), are being developed and assessed as complementary routes for molecular-level characterisation of intra- and inter-cellular properties. The Raman effect results from single photon-molecule, inelastic scattering (UV, visible or near-IR laser light) that causes a change in polarisability and vibrational state of the molecule. The scattered light is assessed spectroscopically, providing a highly-sensitive, detailed molecular-scale fingerprint of the sample. RS is advantageous as it requires little sample preparation, is label-free, and can be used non-destructively. The method has been extensively applied to study biological samples down to single-cell interrogation (e.g., Baldock et al.^12^), with its ability for sensitive molecular-level characterisation being well-established (e.g., Movasaghi et al.^13^). Its use in translational biomedical applications could be further expanded by the development of easy-to-prepare Raman-based assays for stratified phenotyping, created from rigorous data analyses, and verified by reproducibility tests via robust statistical assessment.

As proof-of-concept of this approach, we have applied RS to quantitatively characterise clonal mesenchymal stromal cells (MSCs)^14^ using easy-to-prepare, dried-cell samples. MSCs have been used in clinical trials for cell-based therapies to treat a range of disorders including chronic orthopaedic disorders, such as osteoarthritis and osteoporosis^15^. However, their application in regenerative medicine has been compromised by the inability to reliably and easily identify these cells in highly heterogeneous populations, and to definitively characterise their subtype properties (such as differentiation potential) using cell surface markers, or by profiling^16,17^. Here, we show label-free RS can be used for biomolecular phenotyping and cellular heterogeneity assessment of MSCs, thereby enabling MSC identification within mixed-cell populations and stratification according to shared biological features. The potential to predict specific cell properties, for example, differentiation capability, may also ensue. Such Raman-based measures could be applied as an assay, specific for future therapeutic translation of MSCs in regenerative medicine, and more broadly, to phenotype, and assess heterogeneity in other cell-types pertinent to the fields of biology and biomedicine.

## Results

### Raman spectra show key visual differences across cell lines

The MSC lines in this study were generated by isolating single-cell-derived colonies from a heterogeneous MSC population that was immortalised with human telomerase reverse transcriptase (hTERT)^14^. Four hTERT MSC-lines, Y101, Y102, Y201 and Y202, were chosen with Y101 and Y201 having tri-lineage differentiation capacities, and with Y102 and Y202 being differentiation incompetent (Table S1). The Raman spectra for the hTERT MSCs were tested for cell-type discrimination and the ability to group Y101/Y201 vs. Y102/Y202 due to these groups having previously defined, shared biological features, such as morphologies, immunomodulatory capacities and differentiation competencies^14^. A primary, FACS-sorted MSC sub-set expressing the surface protein, CD317 (CD317+MSCs), which has been shown to have similar features (morphology and immunomodulatory capacity) to the Y102/Y202 non-differentiating cell-lines^14^, was compared as an independent assessment. As MSCs are found in highly heterogeneous mixed-cell populations, a human dermal fibroblast (HDF) cell-line also served as a non-MSC stromal cell control.

**Figure 1:**
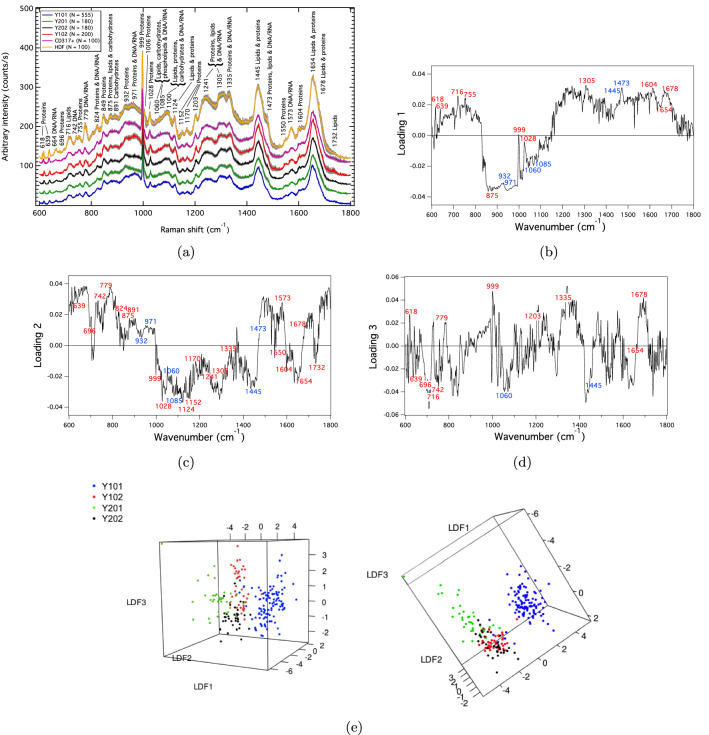
(**a**) Averaged Raman spectra obtained from the cell nuclei of the four hTERT MSCs, primary CD317+MSCs, and HDF cell-populations in the ‘fingerprint’ region (600–1800 cm$^{-1}$), where N is the total number of individual spectra in each spectral average. The SE envelopes associated with each spectrum are shown in grey. Peak positions were averaged across the hTERT MSCs giving the representative values in the figure. Table S2 shows the peak-position ranges for all of the cell types studied in this work, with detailed biomolecular peak-assignments given in Table S3. The spectral resolution was ± 3 cm$^{-1}$. PCA loadings corresponding to the (**b**) PC1, (**c**) PC2 and (**d**) PC3 principal components across the four hTERT MSC-types (cf. Fig. S8), which capture 32%, 22% and 12% of the total variance, respectively. Key points of variance above the background in the PC1 loadings correspond to visual differences seen in the average spectra in (**a**). Univariate analyses involving peak intensity ratios (PIRs) will show that the bands identified in blue have specific discriminatory capabilities. (**e**) PCA-LDA scatterplots for the hTERT MSCs generated by capturing 91% of the PCA variance. Cell-line discrimination using the first three linear discriminant functions (LDFs) is shown. An 84% overall prediction accuracy in determining the hTERT cell-types from these results was achieved using leave-one-out cross-validation.

Averaged Raman spectra and corresponding standard error of the mean (SE) envelopes for the undifferentiated hTERT MSCs (Y101, Y201, Y102 and Y202), primary CD317+MSCs and HDF cell populations are shown in Fig. 1a. Each averaged spectrum is a statistically-converged representation of the cell population with respect to twice the standard deviation (2$\times$SD) and SE (Figs. S1–S7). The representative peak-positions were obtained by averaging across the peak-position values for the hTERT MSC-lines, as these comprise the reference spectra against which other cell types were compared (see Table S2 for the full range of peak positions across all cell-types studied in this work). Biomolecular peak assignments were found from literature comparisons with detailed information about the Raman-active modes given in Table S3.

Key visual differences across the spectra are apparent about the protein band centred at 932 cm$^{-1}$, the protein and DNA/RNA band at 971 cm$^{-1}$, as well in the convolved lipids, phospholipids, carbohydrates and DNA/RNA region resolved at 1060 cm$^{-1}$ and 1085 cm$^{-1}$. The DNA/RNA nucleic acid bands at 779 cm$^{-1}$ and 1573 cm$^{-1}$ are also more distinct in the HDF spectrum compared to how these bands appear in the spectra for the other cell types. The most marked difference across the hTERT MSC-lines occurs in the region containing the 932 cm$^{-1}$ and 971 cm$^{-1}$ bands, corresponding to protein, and protein and DNA/RNA vibrations, respectively.

### Multivariate methods show variances that stratify the hTERT MSCs

Figure 1b–d show the principal component analysis (PCA) loadings for the first three principal components PC1, PC2 and PC3, which account for 32%, 22% and 12% of the variance, respectively. The loading results indicate background differences between the spectra, with PC1 showing distinct features between the four hTERT MSC-lines. The loadings also confirm the key visual differences between the spectra, with PC1 showing marked variance across the hTERT MSCs in the 932 cm$^{-1}$ and 971 cm$^{-1}$ band regions, as well as in the region containing the 1060 cm$^{-1}$ and 1085 cm$^{-1}$ bands.

As PCA was unable to fully discriminate the hTERT MSCs (Fig. S8), a subsequent linear discriminant analysis (PCA-LDA) was performed, which discriminated the four hTERT MSC-types (Fig. 1e). The PCA-LDA results show the Y101 and Y201 cells to be well-separated, with the linear discriminant component LDF3 showing good distinction between the Y102 and Y202 cell-types. The Y101s are most clearly separated from the other three cell-types, which may indicate their ability for spontaneous osteogenic differentiation—this being their most biologically distinct feature. The PCA-LDA results were cross-validated using leave-one-out cross-validation, resulting in an overall 84% prediction accuracy in determining the individual hTERT MSCs (Table S4). A predictive classification of 94.6% for the Y101 and 69.5% for Y201 differentiation-competent cells was obtained. For the Y102 and Y202 differentiation-incompetent cells, there was a 70% prediction accuracy for Y102 and 80.6% prediction accuracy for Y202, with some mixed identification. Hence, the multivariate results confirm the sensitivity of RS to classify the hTERT MSCs according to shared biological features, as well as distinguish with reasonable predictive accuracy, the four MSC-types.

**Figure 2:**
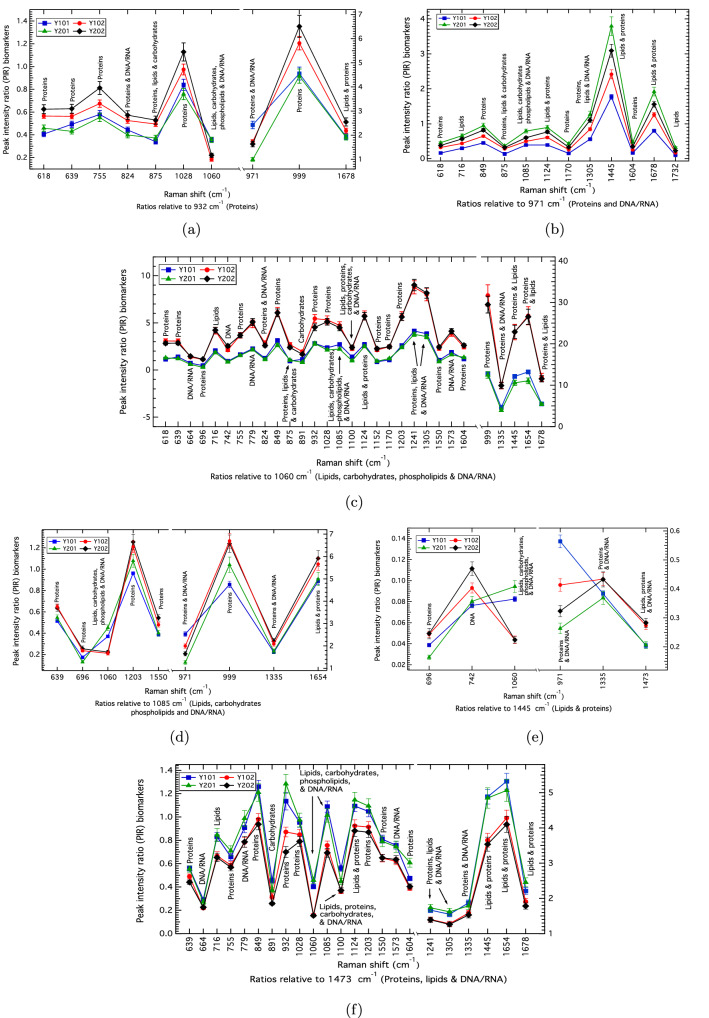
(**a**)–(**f**) hTERT MSC population comparisons (Raman biomarker panels) for the Y101, Y201, Y102 and Y202 cell-lines showing the PIRs that discriminate the Y101/Y201 differentiating vs. Y102/Y202 non-differentiating cell-types, and identify individual hTERT MSC-lines. The uncertainties are the converged propagated standard error of the mean associated with each PIR measurement.

### hTERT MSC ratiometric biomarkers stratify individual cell-lines and those with shared biological features

The Raman spectra were tested for further discriminatory potential using univariate, peak intensity ratio (PIR) analyses. By assessing all-peaks-against-all-peaks in each average hTERT MSC spectrum (Fig. 1a), the PIRs that separate the Y101/Y201 and Y102/Y202 groups, as well as fully discriminate the cell lines, were identified (Fig. 2). Here, ‘fully discriminate’ means complete separation of the PIRs for each MSC-type outside of the SE uncertainties (error bars).

Discriminatory PIR profiles for the biologically-well-defined hTERT MSCs provide sets of panels against which other cell types can be compared. PIRs that fully discriminate the cell lines correspond to proteins, 639/932 (Fig. 2a), as well as those relative to the 971 cm$^{-1}$ band (proteins and DNA/RNA) (Fig. 2b, and also 971/1085 and 971/1445 in Fig. 2d,e, respectively). PIR panels that predominantly distinguish shared biological features of the MSCs by separating the Y101/Y201 and Y102/Y202 differentiating vs. non-differentiating cell-lines were found relative to the 1060 cm$^{-1}$ band corresponding to DNA/RNA, carbohydrates, lipids and proteins (Fig. 2c), and the 1473 cm$^{-1}$ band relating to protein, lipids, DNA/RNA (Fig. 2f). The 932 cm$^{-1}$ (protein), 1085 cm$^{-1}$ (DNA/RNA, carbohydrates, lipids and proteins) and 1445 cm$^{-1}$ (lipids and proteins) panels also show Y101/Y201 and Y102/202 stratification (Fig. 2a,d,e). Across all of these panels, the most discriminatory markers for determining differentiation competency by absolute magnitude differences involve protein and protein-lipid signatures, namely, the 999/1060, 1445/1060 and 1654/1060 PIRs (Fig. 2c), therefore confirming the PCA findings that key differences occur due to protein, lipid and DNA/RNA variations.

**Figure 3:**
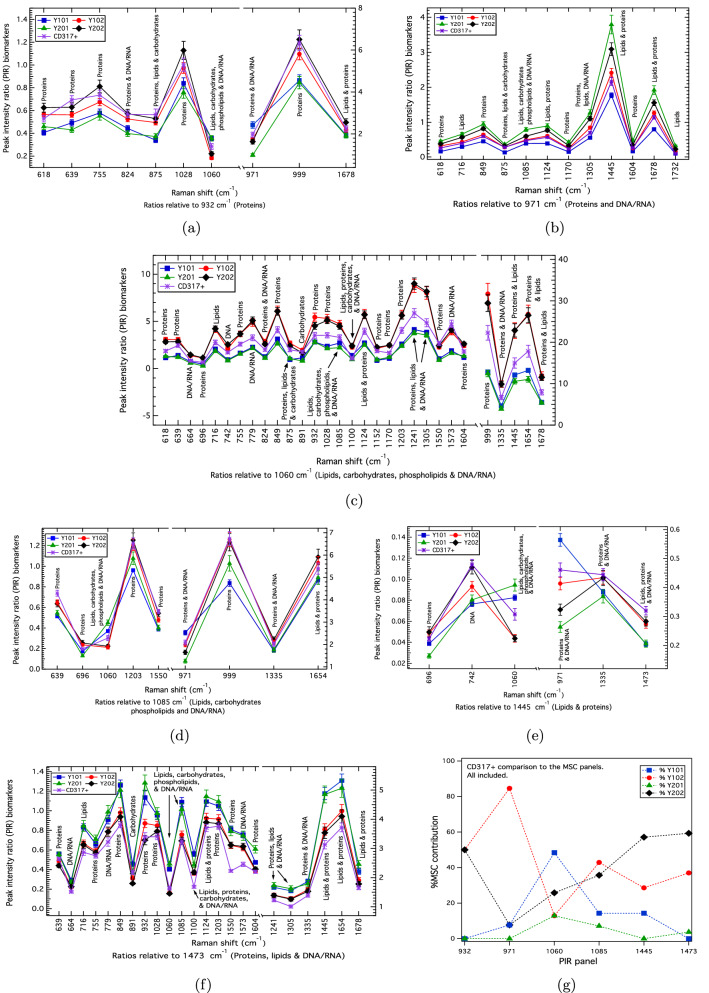
(**a**)–(**f**) PIRs comparing the CD317+MSCs against the hTERT MSC biomarker panels. The uncertainties correspond to the propagated standard error of the mean. The CD317+MSCs show a close match to the Y102/Y202 cell-lines, which aligns with them being biologically-similar as per Ref.^14^. (**g**) KNN-classification across all of the comparison panels confirms a total Y102/Y202 contribution of 82% (Table 1). For the 971 cm$^{-1}$ panel (**b**), which stratifies cell-type, the KNN results in (**g**) shows the CD317+MSCs to be most closely matched at 85% to Y102.

### hTERT MSC Raman biomarker panels classify other cell types

**Figure 4:**
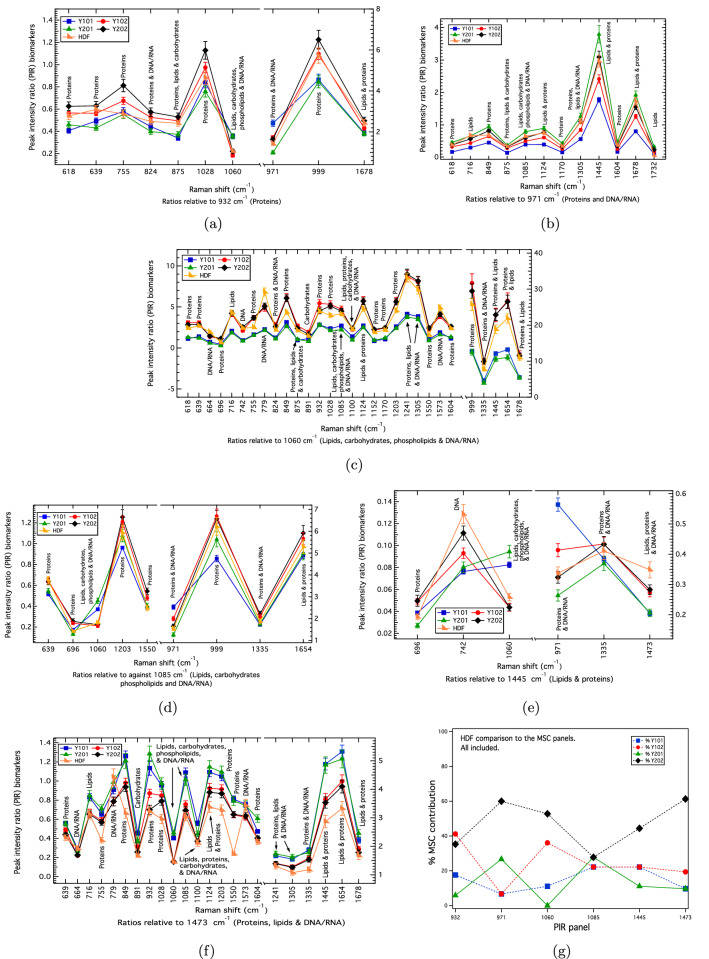
(**a**)–(**f**) PIRs comparing the HDFs against the hTERT MSCs. The uncertainties correspond to the propagated standard error of the mean. For the 971 cm$^{-1}$ panel, which stratifies cell-type, the HDFs are most closely KNN-matched at 60% to Y202 (**b**,**g**). Under standard culturing conditions, HDFs are expected to be differentiation incompetent^21^, which is indicated here by the close (72%) Y102/Y202 match shown in (**g**) assessed across all PIR panels. The relative differences in Y101/Y201 and Y102/202 matches makes the HDFs phenotypically-distinct (Table 1).

The PIRs from FACS-sorted primaries (CD317+MSC) were compared against the hTERT MSC panels, which showed these to be predominantly aligned with the non-differentiating Y102/Y202 cell-lines (Fig. 3). The 971 cm$^{-1}$ PIR panel (discriminatory across all MSC lines) was also closely aligned to Y102 (Fig. 3b). To quantify this match, K-nearest neighbours (KNN) classification was performed (Fig. 3g), which determined an overall 82% classification match against the Y102/Y202 PIRs assessed across all of the comparison panels (Fig. 3a–f and Table 1). For the 971 cm$^{-1}$ panel that stratifies cell-type, the KNN results showed the CD317+MSCs to be most closely matched at 85% to Y102, with an overall 92% match against the Y102/Y202 cell-lines as assessed over all available 971 cm$^{-1}$ PIRs (Fig. 3g). 67% of the 971 cm$^{-1}$ hTERT MSC PIRs were found to be matched within uncertainty (Table S5).

**Table 1:** Cell-type matches (%$_m$) obtained from a KNN model where the closest-distance match between PIR-means in each hTERT MSC panel have been accounted for and then determined across all panels.

Cell type	%$_m$ Y101	%$_m$ Y102	%$_m$ Y201	%$_m$ Y202	%$_m$Y101/Y201	%$_m$ Y102/Y202
CD317+MSCs	14%	43%	4%	39%	18%	82%
HDFs	15%	25%	13%	47%	28%	72%
K72 cell 1	21%	23%	21%	35%	42%	58%
K72 cell 2	44%	13%	29%	14%	73%	27%
K72 cell 3	38%	26%	18%	18%	55%	45%

The hTERT MSC panels were also compared against those determined for adult HDFs as a non-stromal cell control (Fig. 4). MSCs have similar morphologies and properties to fibroblasts, a common type of stromal cell^18,19^. Recent evidence indicates that MSCs and fibroblasts are phenotypically indistinguishable^20^. This finding corresponds with what is known about HDFs kept in standard culturing conditions, namely that they have no potential for tri-lineage differentiation (see for example, Pittenger et al.^21^). Across the panels that correlate to differentiation competency, the HDFs showed overall similarity with the differentiation-incompetent Y102/Y202 cell-lines. KNN-classification against the 971 cm$^{-1}$ individual cell-line panel shows the HDFs to be most closely matched at 60% to Y202, with a 67%-match across both the Y102 and Y202 cell-lines (Fig. 4g). In total, 92% of the 971 cm$^{-1}$ hTERT MSC markers matched within uncertainty (Table S6). An overall 72% KNN-match against the non-differentiating Y102/Y202 cell-lines assessed across the full panel set was obtained (Fig. 4g; Table 1). Compared to the CD317+MSCs, the HDFs have different relative Y101/Y201 and Y102/Y202 %-classification-matchings, hence they are phenotypically distinct (cf. Figs. 3g, 4g, also Table 1).

**Figure 5:**
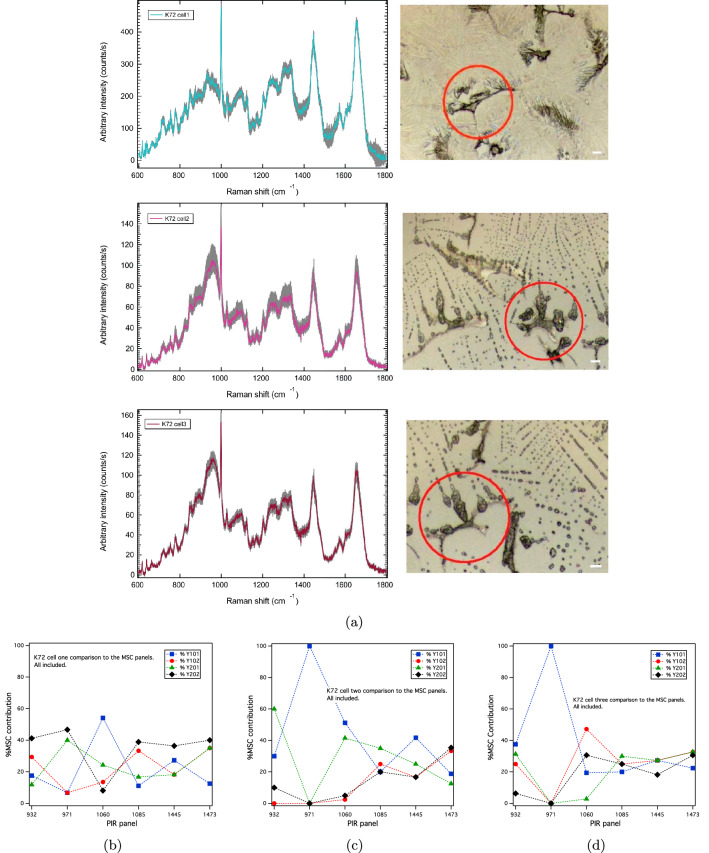
(**a**) Average fingerprints comprising 36 spectra/individual cell map for each of the primary K72 cells circled in the corresponding optical images (cells 1, 2 and 3, respectively). Scale bar = 20 $\upmu$m. (**b**)–(**d**) KNN-classified, % hTERT MSC contributions associated with each individual cell as determined across all PIR markers for each individual panel (Figs. S9–S11). For the 971 cm$^{-1}$ panel, which stratifies cell-type, the K72 cell 1 is most closely matched at 53% to the Y102/Y202 cell-lines. The K72 cells 2 and 3 have 100% matches against Y101. Assessed across all panels and markers, the K72 cell 1 has an overall 58% match against the Y102/Y202 cell-lines, with K72 cells 2 and 3 having overall Y101/Y201 dominance at 73% and 55% matches, respectively (Table 1).

Primary-derived MSC populations are highly heterogeneous groups containing cells of different phenotypes and progenitor characteristics (differentiation capacities)^16,18,19^. Here, we use the hTERT MSC clonal panels to characterise Raman maps of individual primary MSCs (designated K72) obtained from a bone-derived MSC population. Figure 5a shows the converged averaged spectrum from each Raman cell map (see also Figs. S6 and S7), together with the corresponding single-cell optical image. K72 cells 2 and 3 have similar optical features, which are reflected also in spectral similarities, such as the pronounced spectral feature about the 971 cm$^{-1}$ band.

PIR biomarker panels derived from the average spectra for each single-cell map were compared against the hTERT MSC panels (Figs. S9, S10 and S11). KNN-classification against the 971 cm$^{-1}$ individual cell-line panel shows the K72 cell 1 to have a 53% match against the Y102/Y202 cell-lines, with 83% of the total markers for this panel matching within uncertainty (Fig. 5b; Table S7). K72 cells 2 and 3 are significantly Y101-dominant in the 971 cm$^{-1}$ panel having 100%-matches (Fig. 5c,d; Tables S8, S9). Assessed across all of the hTERT MSC panels, the results indicate mixed hTERT features for the individual K72 primary cells (Fig. 5b–d). K72 cell 1 is largely matched with the Y102/Y202 hTERT cell-lines (58% overall), whereas K72 cells 2 and 3 are Y101/Y201 dominant (73% and 55%, respectively) (Table 1). Against the K72 primary MSCs, the HDFs (non-stromal control) also remains phenotypically distinct (cf. Figs. 4g, 5b–d; Table 1).

### PIRs show good reproducibility across replicate experiments

**Figure 6:**
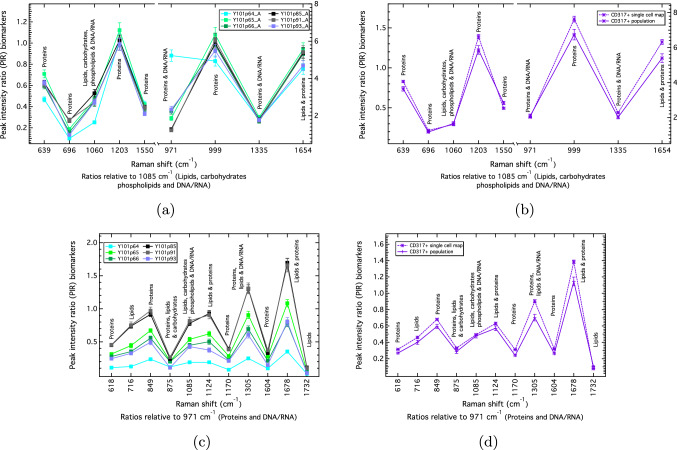
Reproducibility tests for the 1085 cm$^{-1}$ and 971 cm$^{-1}$ PIR panels between experimental replicates for the (**a**), (**c**) Y101 MSC-line showing different passages, p, and (**b**), (**d**) for the FACS-sorted, primary CD317+MSCs showing population and single-cell comparisons. Error bars are the propagated standard errors (SEs).

Reproducibility of the PIRs was tested across experimental replicates. The 1085 cm$^{-1}$ PIR panel, which distinguishes the differentiating hTERT MSCs (Y101/Y201) and non-differentiating hTERT MSCs (Y102/Y202), shows good reproducibility across the hTERT MSC experimental repeats (e.g., Fig. 6a; Fig. S12a–d). Good reproducibility was also obtained for the population and single-cell map comparisons for a Y101 cell-line, CD317+MSCs and K72 primary cells (e.g., Fig. 6b; Fig. S12e–g). A greater number of repeats were performed for the Y101 cell-line (six replicates) due to its ability to spontaneously differentiate^14^. Only one of the replicates (Y101 p64; where p = passage) showed marked differences against the other Y101s in the 1085 panel, specifically for the 971/1085 PIR (Fig. 6a). Differences were also seen in the 971/1085 PIR for the K72 cell 1 and the K72 population against the K72 cells 2 and 3 (Fig. S12g). These differences were represented more specifically in the KNN analysis, which showed the K72 cell 1 to have a greater similarity to Y102/Y202 cell-types, compared to the K72 cells 2 and 3, which were found to be more Y101/Y201-like (Table I) by having variations in the 971/1085 PIR marker. The 971 cm$^{-1}$ panel was examined due to the sensitivities observed with the reproducibility of the 971/1085 PIR, as well as it being distinguishing of the hTERT MSCs; thus, it was expected to show the greatest differences across replicate comparisons. Such differences were indeed apparent for this marker in various degrees in the reproducibility results for the hTERT MSCs, and in the individual cells vs. population reproducibility panels (Fig. 6c; Fig. S13). For example, the Y101s show the greatest differences across experimental repeats (Fig. 6c) compared to the FACS-sorted CD317+MSC population vs. individual cell comparisons (Fig. 6d).

### Raman maps show spatially-resolved heterogeneity linked to differentiation capacities

**Figure 7:**
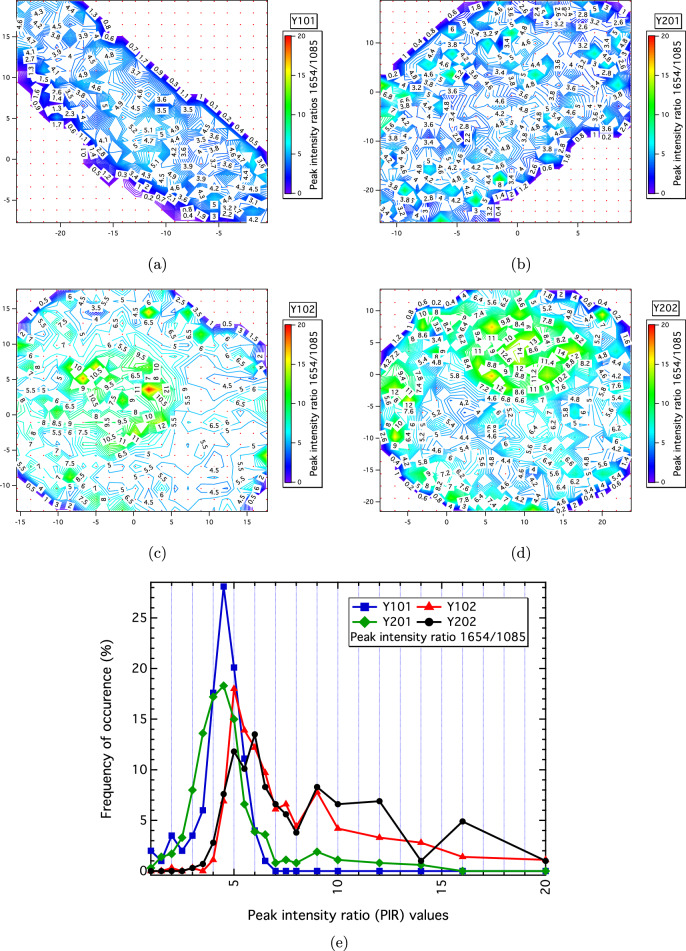
Spatially-resolved Raman maps showing the 1654/1085 PIR that separates the Y101/Y201 (differentiation competent) and Y102/Y202 (differentiation incompetent) hTERT MSC-lines. The differentiation-competent (**a**) Y101 and (**b**) Y201 cells have low, evenly distributed values for this marker, whereas the differentiation-incompetent (**c**) Y102 and (**d**) Y202 cells show concentrated “hot-spots” assumed to be within the cell nucleoli. (**e**) Percentage frequency of occurrence corresponding to the 1654/1085 PIR values measured over the spatially-resolved Raman maps showing discrimination of the Y101/Y201 and Y102/Y202 differentiation competencies. The values were normalised against the number of individual spectra (N) obtained for each cell nucleus [N(Y101) = 199; N(Y102) = 361; N(Y201) = 362 and N(Y202) = 288] and binned as per the points shown.

Raman maps were constructed from spatially-resolved 1654/1085 PIR values as this marker discriminated between the Y101/Y201 (differentiating) and Y102/Y202 (non-differentiating) cell-lines (Fig. 2d). The 1654/1085 PIR also showed good reproducibility in the replicate tests across the average spectra (Fig. 6a,b; Fig. S12). Good reproducibility for this marker was also obtained for the Raman maps due to robustness in the auto-fitting of the 1085 and 1654 bands in each individual spectrum against other bands that were difficult to resolve at single-spectrum-level due to low signal-to-noise. The 1654/1085 PIR shows marked spatial differences in the map results, such as morphological changes (shapes and sizes) between the nuclei for the dried hTERT MSCs (Fig. 7). For example, nuclei of the Y101/Y201-type cells have smaller areas and elongated shapes versus the larger, rounded nuclei of the Y102/Y202-type cells (see Fig. S14 for DAPI results).

The 1654/1085 PIR values in the Raman maps have a relatively even distribution over the Y101/Y201 nuclei compared to the Y102/202 nuclei, which have concentrated regions of higher 1654/1085 PIR-values assumed to be within the nucleoli of the cell (cf. Fig. 7a,b with Fig. 7c,d). Oktar et al. reported a link between nucleoli absence or presence associated with nucleostemin expression, and the resultant differentiation capability (differentiating or non-differentiating, respectively) of MSCs^22^. These findings are commensurate with the Raman maps, namely that the Y101/Y201-type cells, which are differentiation competent, have an absence of well-defined nucleoli, whereas Y102/Y202-type cells, which are differentiation incompetent, have highlighted nucleoli regions via the 1654/1085 PIR marker. The percentage frequency of occurrence of 1654/1085 PIR values normalised against the total number of spectral measurements for each cell nuclei is shown in Fig. 7e, providing a succinct representation of the Raman map results. The Y101/Y201-type cells have lower, more consistent 1654/1085 PIR values across the nuclei, whereas Y102/Y202-type cells have, in general, higher 1654/1085 PIR values with a broader numerical range across the cell nuclei.

### Biomolecularly-stratifed heterogeneity can be quantified via statistical convergence

**Figure 8:**
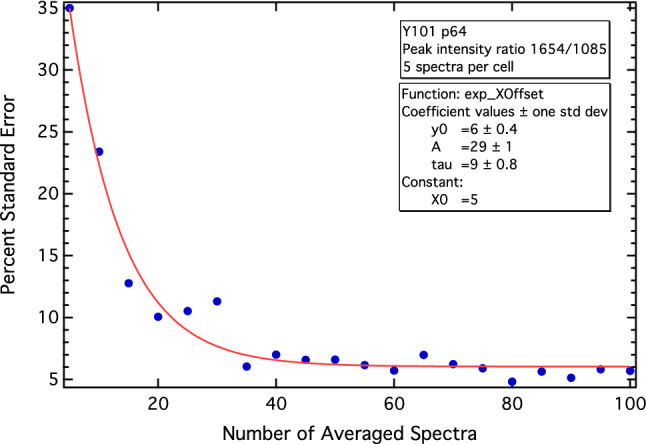
Example percentage standard error (%SE) convergence test showing the “decay” of the %SE associated with the 1654/1085 PIR as a function of increasing, randomly-acquired spectra added to the spectral average. A convergence test such as this can be used to derive a decay constant (tau/$\tau$), interpreted as a quantitative measure of heterogeneity stratified to specific PIRs. The red line shows the fitted exponential function.

Heterogeneity across each cell-line population can be assessed via the rate-of-convergence of the percentage standard error (%SE) as a function of the increasing number of spectra collected for each cell-line added to the fingerprint average, and then stratified to specific PIRs. We assessed the convergence of the two previously studied ratios; 971/1085, which discriminates the four MSC lines, and the 1654/1085 PIR, which shows separation between differentiation-competent Y101/Y201 cell-lines, and the differentiation-incompetent Y102/Y202 cell-lines. For both PIRs, the %SE was calculated and plotted against the number of averaged spectra. An example of a %SE convergence test is shown in Fig. 8. The convergence graphs were fitted with an exponential curve from which a decay constant tau ($\tau$) was derived as a measure of the heterogeneity associated with each population and stratified biomarker (Figs. S15–S20).

**Table 2:** Average decay constants ($\tau$) obtained for the 971/1085 and 1654/1085 PIRs for the different cell populations analysed, together with the number of experiments performed. These summarised results have been extracted from Figs. S15–S20 and Tables S10–S17. A smaller $\tau$ represents a more “rapid decay” of the %SE. For the hTERT MSC-lines (Y101, Y201, Y102 and Y202), the range of $\tau$ values are shown in the brackets. For the other cell types, the uncertainty corresponds to the standard deviation of the exponential fitting since these experiments were performed only once. As the CD317+MSCs were sorted by FACS, the average (Av.) decay rates were also shown for the primaries with their results removed.

		PIR 971/1085	PIR 1654/1085
Sample	Replicates	Av. decay constant ($\tau$)	Av. decay constant ($\tau$)
Y101	6	10.0 (6.2–14.0)	7.7 (4–12)
Y201	2	8.5 (5.7–11.6)	3.5 (2.2–4.6)
Y102	2	8.5 (5.5–12.0)	7.5 (6–9)
Y202	2	11.5 (10.0–12.7)	6.5 (4–8)
Average (hTERTs)	—	9.6	6.3
CD317+MSCs (pop)	1	7 ± 1	8 ± 1
HDF(pop)	1	13 ± 1	8 ± 1
K72(pop)	1	10 ± 1	6.0 ± 0.9
Average (others)	—	10 [11.5 sans CD317+MSCs]	7 [7 sans CD317+MSCs]

Table 2 shows the average decay constants for the cell populations corresponding to the 971/1085 and 1654/1085 PIRs. A smaller $\tau$ indicates a more “rapid decay” of the exponential curve, meaning the PIR has converged to a minimum value of the %SE using a smaller quantity of spectra from across the population. Thus, $\tau$ can be used to stratify and assess heterogeneity for each PIR, with a smaller value indicating a more homogeneous population. For the 971/1085 PIR (protein and DNA/DNA ratio), the average $\tau$ for the hTERT MSCs was found to be smaller than the other cell-types, specifically, the HDF and K72 populations, indicating greater homogeneity. The CD317+MSC primaries, having been sorted by FACS, were the most homogeneous for the 971/1085 marker with a decay constant of 7 ± 1; however, only one experiment was performed. Similar results were obtained for the 1654/1085 PIR (proteins and lipids/DNA ratio), which showed the hTERT MSC $\tau$’s to also be, on average, smaller than the average $\tau$ across the other cell-types.

## Discussion

Understanding cell-level processes that underpin cellular function at the biomolecular-level, including cell-level disease, is paramount to advancing the fields of biological and biomedical research. The role of cellular heterogeneity in phenotypic characterisation, and the ability to easily and tractably quantify and stratify this to the biomolecular-level, remain open challenges. Such understanding, linked to cellular function, could positively impact biomedical applications, for example, in developing cell-level disease treatments through the selection and assessment of cells for downstream testing. Here, we have shown how Raman spectroscopy can be used to create an easy, high-discriminatory and tractable means of stratified phenotyping and quantifying cellular heterogeneity at the biomolecular-level based on representative and robust statistical sampling using an exemplar case of closely-related clonal MSCs.

To demonstrate this application, sets of Raman PIR biomarkers were obtained for four well-characterised clonal hTERT MSC-lines derived from statistically-converged spectral data. For cellular phenotyping, the sensitivity of Raman to characterise these closely-related cell-lines was demonstrated by it eliciting 90 biomarkers across six PIR panels that stratified the clonal hTERT MSC-lines and grouped according to known, shared biological functions (e.g., differentiation competencies) (Fig. 2). The stratifying biomarkers were related to biomolecular differences, namely, to spectral regions sensitive to differentiation-capacity distinction, specifically DNA/RNA, protein and lipid changes. Unsupervised, multivariate PCA and PCA-LDA validated the univariate PIR results with an overall 84% prediction accuracy in individual cell-line identification determined by leave-one-out cross-validation.

To test the sensitivity and accuracy of the hTERT PIR panels for cellular classification, comparisons were made against those determined for primary cell-sorted MSCs (CD317+MSCs) expressing the CD317 protein, which have been shown to be related to the non-differentiating Y102/Y202 hTERT MSCs^14^. Human dermal fibroblasts (HDFs) were also compared as a non-MSC stromal control. Following KNN-classification against the hTERT MSC panels, the CD317+MSCs were correctly identified to be most closely matching to the Y102/Y202 hTERT MSCs (82% match), with the HDFs being phenotypically distinct (Table 1). Previously, we had only shown a very limited set of biomarkers and spectral data, with no replicates^14^. In a further application, the PIR biomarker panels for three randomly-selected, primary MSCs were also assessed and classified against hTERT MSC panels, thereby determining their phenotypic likeness. These findings show the ability to use Raman to generate biomarker panel-sets from biologically well-defined cells (in this case, clonal MSCs) that can be used to identify and characterise key features of more complex and heterogeneous cell-types, such as primary cells of the same type, or against different cells that can be found in mixed, heterogeneous populations.

A benefit of Raman testing, as shown here, is the ability to use this information to understand cell-function and cell-variations via stratified biomolecular differences. In terms of specific biomolecular differences in this exemplar case, the 971 cm$^{-1}$ band, which originates from the vibrations of phosphate monoester groups in phosphorylated proteins and DNA/RNA nucleic acids^13,23^, was shown to stratify the four hTERT MSC-lines (Fig. 2b). Specific, functional pathways relating to this band include increased protein phosphorylation occurring via protein kinase C $\delta$, which has been associated with differentiation capacity^24^, and hyper-phosphorylation of proteins in MSCs in the undifferentiated state detected by phosphoproteomic studies^25^. Protein phosphorylation is also linked to cell signalling that occurs in response to external factors^26^, is regulatory in cell death mechanisms^27^ and, together with downstream DNA/RNA changes, is associated with cellular modifications due to desiccation^28^. Thus, a partial explanation for the significance of the 971 cm$^{-1}$ band in cell-line discrimination may also be biomolecular changes from air-drying or cell death that further enable stratification capability.

Reactive oxygen species production, which leads to changes in proteins, lipids and DNA via phosphorylation and cell desiccation^29^ have also been implicated in differentiation capacity differences in MSCs^30^. Protein sensitivity in the hTERT MSC PIR panels is further supported by proteomic studies^31^, and by protein-specific genetic pathways in MSCs, such as Wnt protein signalling-induced osteo-differentiation^32^. All of these factors may relate to the high number of protein-related Raman markers for MSC characterisation. Lipid-specific membrane compositions pertaining to different cell-lines and their recent implication in MSC differentiation fates^33^ may additionally explain lipid involvement in the PIR panels. The predominance of protein, lipid and DNA signatures is represented across the key bands found at 932 cm$^{-1}$, 971 cm$^{-1}$, 1060 cm$^{-1}$, 1085 cm$^{-1}$, 1445 cm$^{-1}$ and 1473 cm$^{-1}$ (Fig. 1). The ability to link phenotypic expression to underlying function, as demonstrated here, which stem from stratified biomolecular differences, could also be applied more broadly to other cell systems across the fields of biology and biomedicine, for example, to study cell-level disease processes, and in applications involving cell selection, diagnostics, or to monitor cell response in the development of targeted treatments.

A key aim of this work was to propose a means by which cellular heterogeneity could be biomolecularly quantified and stratified. In this respect, we have shown various ways by which statistically-converged Raman data can be easily used for this purpose. In our example case, the Raman biomarker maps of the hTERT MSCs, which contained a statistically-representative amount of spatially-resolved point spectra, provided further phenotypic distinction for the 1654/1085 PIR biomarker that stratified the differentiating and non-differentiating (Y101/Y201 vs. Y102/Y202) MSCs types (Fig. 7). These maps also highlighted morphological differences in the sizes of the nuclei found to be in general agreement with DAPI experiments of the nuclei perimeters and areas of the hTERT MSCs (Fig. S14). The spatially-resolved Raman information therefore provides greater detail by which spatial heterogeneity linked to functional differences can be assessed.

Reproducibility tests for the PIR biomarkers across replicates also showed how heterogeneity could be stratified and quantified to individual PIR markers. Although both the 971/1085 PIR (which stratified cell-type) and 1654/1085 PIR (which stratified cell-function, e.g., differentiation capacity) showed very good replicate reproducibility, the former indicated greater variation (i.e., heterogeneity) across the replicates (Fig. 6; Figs. S12 and S13). We believe these variations to be primarily related to the overall differences in the MSC differentiation-potential across the hTERT MSCs. Statistical convergence tests, where the %SE was converged as a function of the increasing number of spectra in the spectral average, confirmed the 971/1085 PIR, which stratifies cell-type, to also have greater biomolecularly-resolved heterogeneity compared to the 1654/1085 PIR across the cell-line populations. These comparisons were made via a proposed heterogeneity-measure determined from the *rate* of convergence ($\tau$) (Fig. 8). If linked to cell-fate and biological function, biomolecularly-stratified heterogeneity measures, such as these, could be used to engineer more phenotypically-homogenous populations of cells for specific purposes. For the MSCs, this could mean selecting the best choice of cells for specific and *directed* differentiation, ensuring sufficient MSCs within a population to produce large-scale and targeted tissue development; another issue limiting their biomedical translation and therapeutic purpose.

In summary, we obtained Raman PIR panels comprising a complete set of distinct biomarkers for clonal hTERT MSCs that correlate to what is known about the differentiation competencies of each cell-type, with a sub-set that fully distinguishes the cell lines. Using these panels, we were able to classify and identify other cell types, and via statistical convergences, assess and quantify biomolecular-level stratified heterogeneities. Compared to more complex classification methods, which require substantial sample preparation, we propose a means by which a statistically robust, univariate, Raman spectroscopy assay can be derived. Such an assay would be label-free, rich in information for precision stratification, and easy to produce and measure. Using these methods, Raman-based assays could then be derived for other cell systems (both dried and live cell, and for tissues), seeing broad application within the fields of biology and biomedicine, and overcoming current limitations in obtaining quantitative and biomolecularly-stratified phenotyping and assessment of heterogeneity linked to cell function.

### Methods

All methods were carried out in accordance with relevant guidelines and regulations under approval from the University of York Biology Ethical Committee and NHS Local Research Ethics Committee.

### Cell culturing

Primary MSCs were obtained from tissue samples removed during routine joint replacement therapy. Informed consent was obtained from all subjects and all methods were carried out in accordance with relevant guidelines and regulations. Protocols for obtaining and characterising the hTERT MSC lines and acquiring the CD317+ fraction from a primary MSC population using FACS can be found in Ref.^14^. Human dermal fibroblasts (HDFs) were purchased from Cascade Biologics (Life Technologies). Cells were grown at 37 $^{\circ }$C and 5% CO$_{2}$ in Dulbecco’s Modified Eagle’s Medium (DMEM) with 1% penicillin–streptomycin. Fetal bovine serum (FBS) was supplemented at 10% for the hTERT MSC clonal lines and the HDFs, and at 15% for the primary MSCs (CD317+MSCs and K72). At $\sim$70% confluence, the growth medium, including any non-adherent cells, were removed and the cells rinsed with 10% phosphate buffered saline solution (PBS). The cells were then detached from the flask by adding trypsin-EDTA, harvested, and then re-seeded at 1/4 of their total amount. After these processes, we would expect apoptotic cells that are known to detach from the extracellular matrix^34–36^ to be in the cell media suspension and to not initially seed or re-seed after trypsination. The passages (p) were: Y101 (p64, p65, p66, p85, p91, p93), Y102 (p66, p79), Y201 (p60, p63), Y202 (p76, p80), CD317+MSC (max. p9), K72 (p3–p9) and HDF (p20–p50).

### DAPI experiments

The four hTERT MSC-lines (Y101, Y102, Y201 and Y202) were established and maintained as described by James et al.^14^. Cells at 70% confluency were briefly trypsinised; 125,000 cells were applied to 13 mm number 1.5 glass coverslips (Scientific Laboratory Supplies) and allowed to adhere for 4 h. Cells were fixed with 3% paraformaldehyde (Park Scientific) for 20 min and gently washed 3$\times$ with PBS. DAPI (2 $\upmu$g/ml) was applied for 10 min before an additional 3$\times$ washes in PBS. Coverslips were inverted into vectashield mounting media for fluorescence (Vector Laboratories) and imaged at 10$\times$ magnification on a Leica DM IRB microscope coupled with a Leica DC500 camera. Images were processed in Image J v1.49 (National Institutes of Health, USA)^37^. Briefly, the RGB images were threshold adjusted and particles were analysed with area and perimeter measurements being recorded. Images were screened for validity excluding part-cells at the image edge, small objects that were not cells, and large objects that were two or more cells that could not be discriminated. Data from at least 67 cells that met the inclusion criteria were processed in GraphPad Prism v6.07 with calculations for average area and perimeter.

### Sample preparation for Raman spectroscopy

Harvested cell suspensions were centrifuged for 5 min at 1200 rpm to remove the trypsin supernatant and then resuspended in the growth medium. An evenly distributed single-cell layer required for independent cell measurements was achieved by gentle pipette agitation of the cell solution followed by seeding at a density of $5 \times 10^5$ cells per calcium fluoride (CaF$_2$) microscope slide (75 mm $\times$ 25 mm $\times$ 1 mm) (Fig. S21). The slides were then incubated for 24-h in a Petri dish containing 15 mL of DMEM supplemented with 15% FBS, 1% penicillin streptomycin and 0.1% Amphotericin B to allow cell attachment. To remove cell-cycle dependency^38^, cells were synchronised to the G0-state via nutritional deprivation by replacing the culture medium with a 0.5% FBS-concentrated DMEM solution and then re-incubating for a 24-h^39^. The microscope slide was then removed from the medium, rinsed twice with PBS and air-desiccated for 30 min in a fume cupboard. Against fixative solutions, air-drying and desiccation provide good signal intensity and preservation of cell components in the Raman spectrum^40^. To ensure the highest quality results without time-dependent degradation, the spectra were collected from the samples immediately upon being dried.

### Raman spectroscopy measurements

Raman point spectra were collected using an HORIBA XploRA micro-Raman in confocal setting (100 $\upmu$m pinhole), with 200 $\upmu$m slit, 532 nm laser wavelength at 3.5 mW laser-power and 2400 lines/mm diffraction grating. An 100$\times$ (NA = 0.9) objective was used resulting in a diffraction-limited laser spot size of $\sim$ 1 $\upmu$m. The spectral resolution was 3 cm$^{-1}$. Spectra were obtained using 45 s laser exposure averaged over two spectral acquisitions. To prevent laser damage, the cells were monitored during real-time acquisition to ensure no spectral changes, and optical inspection was also performed after each measurement. Five random spectra were obtained per optically well-defined and demarcated nucleus of randomly selected cells per cell population. In the case of label-free Raman measurements, no cell viability assays could be used to identify healthy and viable prior to air-dried desiccation. Hence, in addition to cell-culturing processes that best removed apoptotic cells due to their detachment (inability to seed), we also excluded from the random selection process in the dried-state, those that had morphological features associated with apoptotic cells. Namely, we only sampled dried cells with regular, intact edges and surfaces (no evidence of blebbing, excessive thickening of the nuclear membrane or significant loss of membrane integrity/leakage) and having intact cellular structure/nucleus (no fractionation) (see Refs.^34–36^, which describe morphological apoptotic-cell features). Convergence of the average spectrum, twice the standard deviation (2$\times$SD) and standard error of the mean (SE) for increasing numbers of spectra ensured the data were statistically-representative of the dried-cell state (nuclei and cytoplasm). For the hTERT MSCs: Y101 = 555 spectra (six experiments, with several repeats due to its ability to spontaneously differentiate), Y201 and Y202 = 180 spectra (two experiments) each, Y102 = 200 spectra (two experiments). For the other cell lines: HDFs = 100 spectra (one experiment); CD317+MSCs and K72 populations = 100 spectra (one experiment) each. For the K72 MSC primaries, 36 spectra/nucleus were obtained from three cells, randomly selected. Spatially-resolved Raman maps (360–484 point spectra/map) were also obtained from single cells randomly chosen in each hTERT MSC-line population.

### Principal component and linear discriminant analyses

PCA and PCA-LDA were performed on the Raman results for the hTERT MSCs using R (version 3.3.0)^41^. Each spectrum was first interpolated using code written in IGOR Pro 6.32 to ensure the same wavenumber increments (hence PCA channels) across the spectra. The spectra had minimal background removal through linear baselining as this has previously been shown to discriminate cell phenotypes^42,43^. The five spectra obtained for each single cell nuclei were then averaged using IGOR Pro, area-normalised and cubic-spline-smoothed, using 0.65 for the smoothing parameter. Spectral processing was performed using the *Raman tool set* package^44^. PCA and LDA plots were produced, as well as the corresponding PCA loadings, followed by leave-one-out cross-validation. 18 PCs, accounting for 91% of the variance, were incorporated into the LDA as per the protocol in Ref.^45^.

### Peak intensity ratio (PIR) analyses

Each spectrum was linear baseline-corrected from the first to the last spectral point using the *Raman tool set* package^44^ and then averaged using IGOR Pro. To determine a statistically-representative number of spectra, convergence tests were performed on the 2$\times$SD and SE as a function of the increasing number of spectra in the spectral average (Figs. S1–S7). Gaussian peak-fitting was performed across linear-baselined spectral windows using the IGOR Pro Multipeak Fitting 2 function on each average spectrum, with an auto peak-fitting code used for the spatially-resolved maps. PIRs were calculated from the intensities determined from the peak fittings, with the uncertainties obtained from the propagated SEs. Raman maps were generated from spatially-determined PIRs across the nucleus using an auto-generating procedure developed in IGOR Pro. A second convergence test was developed for the %SE for the PIRs (Figs. S15–S20). Strict convergence of the statistical quantities ensured experimental variability was fully accounted for as per the consistency in results across normalised and non-normalised spectra (e.g., Fig. S22).

### PIR panels and KNN classification

PIRs, plus the propagated SE uncertainties, were determined for all-peaks-against-all-peaks for each hTERT MSC-line. From these PIRs, those that showed Y101/Y201 differentiating, and/or Y102/Y202 non-differentiating stratification, or individual hTERT MSC-line stratification outside of the propagated SE, were used to create the hTERT MSC comparison panels against which the PIRs from other cell-types (population-level and individual cells) were compared. To quantify a %-match to each of the hTERT MSC-lines, a KNN classification model was devised, whereby the closest nearest neighbour match (both within and outside of SE-uncertainty) for each PIR per panel was determined (see e.g., Ref.^46^ for a description of the KNN algorithm).

## Supplementary Information

Supplementary Information.

## Data Availability

The datasets generated during and/or analysed during this study are available by request from Y.H. from the University of York repository 10.15124/597ad871-7b1f-4ab3-9d5d-93a4235b15f8.

## References

[1] Stockholm D, Benchaouir R, Picot J, Rameau P, Neildez TMA, Landini G, Laplace-Builhe C, Paldi A (2007). The origin of phenotypic heterogeneity in a clonal cell population in vitro. PLoS One.

[2] Avery SV (2006). Microbial cell individuality and the underlying sources of heterogeneity. Nat. Rev. Microbiol..

[3] Chang HH, Hemberg M, Barahona M, Ingber DE, Huang S (2008). Transcriptome-wide noise controls lineage choice in mammalian progenitor cells. Nature.

[4] Cooper GM (2002). The Cell: A Molecular Approach.

[5] Cohen AA, Geva-Zatorsky N, Eden E, Frenkel-Morgenstern M, Issaeva I, Sigal A, Milo R, Cohen-Saidon C, Liron Y, Kam Z, Cohen L, Perzov N, Alon U (2008). Dynamic proteomics of individual cancer cells in response to a drug. Science.

[6] Fernandes RL, Nierychlo M, Lundin L, Pedersen AE, Tellez PEP, Dutta A, Carlquist M, Bolic A, Schapper D, Brunetti AC, Helmark S, Heins AL, Jensen AD, Nopens I, Rottwitt K, Szita N, van Elsas JD, Nielsen PH, Martinussen J, Sorensen SJ, Lantz AE, Gernaey KV (2011). Experimental methods and modeling techniques for description of cell population heterogeneity. Biotechnol. Adv..

[7] Katsanis SH, Katsanis N (2013). Molecular genetic testing and the future of clinical genomics. Transl. Genet..

[8] Gawad C, Koh W, Quake SR (2016). Single-cell genome sequencing: Current state of the science. Nat. Rev. Genet..

[9] Lo P-K, Zhou Q (2018). Emerging techniques in single-cell epigenomics and their applications to cancer research. J. Clin. Genomics.

[10] Lowe WL, Reddy TE (2015). Genomic approaches for understanding the genetics of complex disease. Genome Res..

[11] Li SC, Tachiki LML, Kabeer MH, Dethlefs BA, Anthony MJ, Loudon WG (2014). Cancer genomic research at the crossroads: Realizing the changing genetic landscape as intratumoral spatial and temporal heterogeneity becomes a confounding factor. Cancer Cell Int..

[12] Baldock SJ, Talari ACS, Smith R, Wright KL, Ashton L (2019). Single-cell Raman microscopy of microengineered cell scaffolds. J. Raman Spectrosc..

[13] Movasaghi Z, Rehman S, Rehman IU (2007). Raman spectroscopy of biological tissues. Appl. Spectrosc. Rev..

[14] James S, Fox J, Afsari F, Lee J, Clough S, Knight C, Ashmore J, Ashton P, Preham O, Hoogduijn M, Ponzoni RAR, Hancock Y, Coles M, Genever P (2015). Multiparameter analysis of human bone marrow stromal cells identifies distinct immunomodulatory and differentiation-competent subtypes. Stem Cell Rep..

[15] Fox JM, Genever PG (2014). Use of adult stem cells for orthopedic regenerative medicine applications. Cell Tissue Transpl. Ther..

[16] Bianco P, Riminucci M, Gronthos S, Robey PG (2001). Bone marrow stromal stem cells: Nature, biology and potential applications. Stem Cells.

[17] Roberts S, Genever P, McCaskie A, De Bari C (2011). Prospects of stem cell therapy in osteoarthritis. Regen. Med..

[18] Russell KC, Phinney DG, Lacey MR, Barrilleaux BL, Meyertholen KR, O’Connor KC (2000). In vitro high-capacity assay to quantify the clonal heterogeneity in trilineage potential of mesenchymal stem cells reveals a complex hierarchy of lineage commitment. Stem Cells.

[19] Pevsner-Fischer M, Levin S, Zipori D (2011). The origins of mesenchymal stromal cell heterogeneity. Stem Cell Rev. Rep..

[20] Denu RA, Nemcek S, Bloom DD, Goodrich AD, Kim J, Mosher DF, Hematti P (2016). Fibroblasts and mesenchymal stromal stem cells are phenotypically indistinguishable. Acta Haematol..

[21] Pittenger MF, Mackay AM, Beck SC, Jaiswal RK, Douglas R, Mosca JD, Moorman MA, Simonetti DW, Craig S, Marshak DR (1999). Multilineage potential of adult human mesenchymal stem cells. Science.

[22] Oktar PA, Yildirim S, Balci D, Can A (2011). Continual expression throughout the cell cycle and downregulation upon adipogenic differentiation makes nucleostemin a vital human MSC proliferation marker. Stem Cell Rev. Rep..

[23] Meurens M, Wallon J, Tong J, Noel H, Haot J (1996). Breast cancer detection by Fourier transformed infrared spectrometry. Vib. Spectrosc..

[24] Lee S, Cho H-Y, Bui HTT, Kang D (2014). The osteogenic or adipogenic lineage commitment of human mesenchymal stem cells is determined by protein kinase C delta. BioMed Central Cell Biol..

[25] Lo T, Tsai C-F, Shih Y-RV, Wang Y-T, Lu S-C, Sung T-Y, Hsu W-L, Chen Y-J, Lee OK (2012). Phosphoproteomic analysis of human mesenchymal stromal cells during osteogenic differentiation. J. Proteome Res..

[26] Hunter T (2012). Why nature chose phosphate to modify proteins. Philos. Trans. R. Soc. B.

[27] Gjertsen BT, Doskeland SO (2009). Protein phosphorylation in apoptosis. Biochim. Biophys. Acta.

[28] Huang Z, Tunnacliffe T (2004). Response of human cells to desiccation: Comparison with hyperosmotic stress response. J. Physiol..

[29] Kranner I, Birtić S (2005). A modulating role for antioxidants in desiccation tolerance. Integr. Comp. Biol..

[30] Li Q, Gao Z, Chen Y, Guan M-X (2017). The role of mitochondria in osteogenic, adipogenic and chondrogenic differentiation of mesenchymal stem cells. Protein Cell.

[31] Billing AM, Hamidane HB, Dib SS, Cotton RJ, Bhagwat AM, Kumar P, Hayat S, Yousri NA, Goswami N, Suhre K, Rafi A, Graumann J (2016). Comprehensive transcriptomic and proteomic characterization of human mesenchymal stem cells reveals source specific cellular markers. Sci. Rep..

[32] Brun J, Fromigué O, Dieudonné F-X, Marty C, Chen J, Dahan J, Wei Y, Marie PJ (2013). The LIM-only protein FHL2 controls mesenchymal cell osteogenic differentiation and bone formation through Wnt5a and Wnt10b. Bone.

[33] Levental KR, Surma MA, Skinkle AD, Lorent JH, Zhou Y, Klose C, Chang JT, Hancock JF, Levental I (2017). $$\omega$$-3 polyunsaturated fatty acids direct differentiation of the membrane phenotype in mesenchymal stem cells to potentiate osteogenesis. Sci. Adv..

[34] Häcker G (2000). The morphology of apoptosis. Cell Tissue Res..

[35] Taylor RC, Cullen SP, Martin SJ (2008). Apoptosis: Controlled demolition at the cellular level. Nat. Rev. Mol. Cell Biol..

[36] Ziegler U, Groscurth P (2004). Morphological features of cell death. News Physiol. Sci..

[37] Rasband, W. S. ImageJ, U.S. National Institutes of Health, Bethesda. https://imagej.nih.gov/ij/ (1997–2018).

[38] Matthaus C, Bird B, Miljkovic M, Chernenko T, Romeo M, Diem M (2008). Infrared and Raman microscopy in cell biology. Methods in Cell Biol..

[39] Achille V, Mantelli M, Arrigo G, Novara F, Avanzini MA, Bernardo ME, Zuffardi O, Barosi G, Zecca M, Maccario R (2011). Cell-cycle phases and genetic profile of bone marrow-derived mesenchymal stromal cells expanded in vitro from healthy donors. J. Cell. Biochem..

[40] Mariani MM, Lampen P, Popp J, Wood BR, Deckert V (2009). Impact of fixation on in vitro cell culture lines monitored with Raman spectroscopy. Analyst.

[41] R: A Language and Environment for Statistical Computing, R Core Team, R Foundation for Statistical Computing, Vienna. https://www.R-project.org.

[42] Pudlas M, Berrio DAC, Votteler M, Koch S, Thude S, Walles H, Schenke-Layland K (2011). Non-contact discrimination of human bone marrow-derived mesenchymal stem cells and fibroblasts using Raman spectroscopy. Med. Laser Appl..

[43] Lieber CA, Kabeer MH (2010). Characterization of pediatric Wilms’ tumor using Raman and fluorescence spectroscopies. J. Pediatr. Surg..

[44] Candeloro P, Grande E, Raimondo R, Mascolo DD, Gentile F, Coluccio ML, Perozziello G, Malara N, Francardi M, Frabizio ED (2013). Raman database of amino acid solutions: A critical study of extended multiplicative signal correction. Analyst.

[45] Chan JW, Lieu DK, Huser T, Li RA (2009). Label-free separation of human embryonic stem cells and their cardiac derivatives using Raman spectroscopy. Anal. Chem..

[46] Zhang Z (2016). Introduction to machine learning: k-nearest neighbors. Ann. Transl. Med..

