# Supplementary material for: Biomolecular phenotyping and heterogeneity assessment of mesenchymal stromal cells using label-free Raman spectroscopy
Source: Sci Rep. 2021 Feb 23;11:4385. doi: 10.1038/s41598-021-81991-1 (PMC7902661; doi:10.1038/s41598-021-81991-1)
Supplement: Supplementary file 1 — Supplementary Information. [file 41598_2021_81991_MOESM1_ESM.pdf]

Supplementary Information

Biomolecular phenotyping and heterogeneity assessment of mesenchymal stromal cells using label-free Raman spectroscopy

R. A. Rocha,[1,2] J. M. Fox,[3,4] P. G. Genever,[3,4] and Y. Hancock[1,4,5,6]

[1] *Department of Physics, University of York, Heslington, York, U.K., YO10 5DD*

[2] *Federal University of Technology—Paraná, Campus Dois Vizinhos, Paraná, Brazil, 85660-000*

[3] *Department of Biology, University of York, Heslington, York, U.K., YO10 5DD*

[4] *York Biomedical Research Institute, University of York, Heslington, York, U.K., YO10 5DD*

[5] *York Cross-disciplinary Centre for Systems Analysis, University of York, Heslington, York, U.K., YO30 5GG*

[6] *School of Cancer & Pharmaceutical Sciences, Faculty of Life Sciences and Medicine, King's College London, London, U.K., SE19RT*

I. SUPPLEMENTARY TABLES

A. hTERT MSC-lines

TABLE S1. Differentiation capacities and CD317 protein expression in the four hTERT MSC-lines [S1].

hTERT MSC-line	Osteogenic	Adipogenic	Chondrogenic	CD317 expression
Y101	Yes (spontaneous)	Yes	Yes	No
Y201	Yes (induced)	Best	Yes	No
Y102	Extremely limited	Limited	No	Yes
Y202	Extremely limited	Limited	No	Yes

B. Raman peak information

TABLE S2: Peak ranges, mean peak positions, standard deviations (SD), standard errors of the mean (SE), and Δ_{max} peak-range values. The ranges shown take into account all of the cell types studied in this work. All units are in cm^{-1}.

**Peak Range**	**Mean Position**	**SD**	**SE**	Δ_{max}
614.6—618.3	617.2	1.2	0.4	3.7
636.6—639.4	638.5	1.0	0.3	2.8
661.6—665.0	663.8	1.1	0.4	3.5
691.3—696.1	695.3	2.1	0.7	4.8
713.4—716.8	716.2	1.4	0.5	3.4
740.4—742.9	742.0	1.0	0.3	2.5
752.9—755.5	754.4	1.0	0.3	2.6
773.2—778.9	777.9	1.8	0.6	5.7
821.2—823.8	823.1	1.1	0.4	2.6
846.7—849.4	848.4	0.9	0.3	2.7
873.8—875.6	874.8	0.6	0.2	1.9
889.5—892.1	890.3	1.3	0.4	2.6
929.4—933.5	932.0	1.3	0.4	4.1
964.8—971.9	969.3	2.6	0.9	7.1
996.8—999.5	998.6	1.0	0.3	2.7
1003.6—1006.3	1005.6	1.3	0.4	2.7
1025.7—1028.4	1027.3	1.0	0.3	2.7
1058.2—1061.4	1058.9	2.1	0.7	3.2
1083.0—1086.5	1084.0	3.1	1.0	3.5
1097.4—1100.4	1099.2	1.1	0.4	3.0
1120.6—1123.8	1123.0	1.2	0.4	3.2
1150.1—1152.7	1151.7	1.1	0.4	3.2
1166.4—1170.1	1169.7	1.6	0.5	3.7
1200.5—1203.3	1202.5	1.0	0.3	2.8
1237.7—1241.7	1240.6	1.6	0.5	4.0
1300.2—1306.0	1304.8	2.2	0.7	5.8
1332.7—1336.1	1334.6	1.3	0.4	3.4
1443.4—1446.4	1445.8	1.1	0.4	3.0

Continued on next page

TABLE S2 (continued)

**Peak Range**	**Mean Position**	**SD**	**SE**	Δ_{max}
1466.2—1478.0	1473.4	3.4	1.1	11.8
1546.6—1550.4	1550.0	2.1	0.7	3.8
1570.5—1573.8	1572.4	1.0	0.3	3.3
1601.7—1607.8	1604.5	1.9	0.6	6.1
1653.8—1655.5	1654.3	0.6	0.2	1.7
1676.6—1679.0	1678.0	0.7	0.2	2.4
1736.5—1739.7	1737.8	1.1	0.4	3.2

C. Raman band assignments for biological cells

TABLE S3: Biomolecular peak-assignments (peak ranges, general and detailed assignments) obtained from the literature.

**Peak Range (cm^{-1})**	**General assignment**	**Detailed biomolecular assignments**
618–623	Proteins	C-C twist of proteins [S2]<br>Phenylalanine C-C twist [S3]
640–645	Proteins	C-S stretch & C-C twist of tyrosine [S2]<br>C-C twist of tyrosine [S3]
666–678	DNA/RNA	Guanine and thymine ring breathing mode [S2, S3]<br>Tyrosine-G backbone in RNA [S2]
700	Proteins	Aminoacid methionime ν(C-S) *trans* [S2]
717–719	Lipids	$CN^+(CH_3)_3$ stretch in lipids [S2–S4]<br>C-N membrane phospholipid head stretch [S2, S5]<br>Adenine nucleotide peak [S2]
746	DNA	Thymine ring breathing mode [S2, S6]
755–760	Proteins	Tryptophan ring breathing mode [S2–S5, S7]
774–788	DNA/RNA	Uracil ring breathing mode [S2]<br>Uracil, Cytosine and thymine ring breathing modes [S3]<br>DNA/RNA O-P-O symmetric stretch [S6]<br>Uracil, cytosine, and thymine overlapping with O-P-O stretch in DNA [S5, S7]
823–830	Proteins & DNA/RNA	Tyrosine out-of-plane ring breathing and O-P-O stretch in DNA [S2–S4]<br>DNA O-P-O asymmetric stretch [S6]<br>Proline and hydroxyproline, out-of-plane tyrosine ring breathing, DNA/RNA asymmetric O-P-O stretch [S5]
850–853	Proteins	Tyrosine ring breathing [S2–S4]<br>Protein related peak [S7]<br>C-C stretch in collagen, tyrosine ring breathing, glycogen, polysaccharides C-O-C stretch [S5]
874–877	Proteins, lipids<br>& carbohydrates	Proteins C-C stretch, antisymmetric vibrations of choline $N(CH_3)_3$ [S2]<br>C-C-N+ symmetric stretch (lipids) and C-O-C ring (carbohydrates) [S3]

Continued on next page

TABLE S3 (continued): Biomolecular peak-assignments

**Peak Range (cm^{-1})**	**General assignment**	**Detailed biomolecular assignments**
		Hydroxyproline C-C stretch in collagen, tryptophan ring deformation, choline asymmetric stretch in phospholipids and C-O-C stretch in carbohydrates [S5]
889–891	Carbohydrates	Saccharide band, methylene and C-C skeletal [S2]
915–938	Proteins	Skeletal C-C stretch α-helix [S2, S7, S8],<br>C-C backbone stretch α-helix and C-O-C glycogen [S3–S5]
968–975	Proteins & DNA/RNA	Phosphate monoester groups of phosphorylated proteins and cellular nucleic acids [S2, S9]<br>C-O-P phosphodiester residue [S10]<br>Ribose (DNA/RNA) [S11]
1000–1005	Proteins	Phenylalanine ring breathing [S2, S3, S5, S7, S8, S10–S12]
1029–1033	Proteins	Phenylalanine ring breathing [S2–S4, S7]<br>Collagen, keratin, C-N stretch in proteins, Phe C-H in plane bending, phospholipids and polysaccharides [S5]
1060–1095	Lipids, carbohydrates,<br>phospholipids & DNA/RNA	DNA PO^{-2} backbone stretch, lipids chain C-C stretch, carbohydrates C-O and C-C stretch [S2, S3]<br>Peak indicative of phospholipids [S8]
1083–1095	Lipids, carbohydrates,<br>phospholipids & DNA/RNA	DNA PO^{-2} backbone stretch and C-C gauche in lipids [S2, S6]<br>DNA PO^{-2} symmetric stretch [S10]<br>DNA PO^{-2} stretch, lipids chain C-C stretch, carbohydrates C-O and C-C stretch [S3]
1095–1100	Lipids, proteins	DNA PO^{-2} stretch [S2]<br>C-C vibration of gauche bonded chain [S2]<br>Peak indicative of lipids [S8]<br>DNA PO^{-2} stretch, lipids chain C-C stretch, carbohydrates C-O and C-C stretch [S3, S4]
1123–1128	Lipids & proteins	Protein C-N stretch [S2–S4]<br>ν(C-C) skeletal backbone in lipid (transconformation) [S2, S6]<br>C-C stretch in proteins and C-O stretch in carbohydrates [S2]<br>Peak indicative of phospholipids [S8], lipids [S11]

Continued on next page

TABLE S3 (continued): Biomolecular peak-assignments

**Peak Range (cm^{-1})**	**General assignment**	**Detailed biomolecular assignments**
1152–1158	Proteins	C-C and C-N stretch in proteins [S2–S4]<br>Carotenoids [S2]
1170–1176	Proteins	C-H in plane bend of Tyrosine [S2, S3]<br>Protein-related peak [S7]
1200–1209	Proteins	Tyrosine and Phenylalanine [S2, S3, S6]<br>Phenylalanine and tryptophan [S4]<br>CH_2 wagging of glycine and proline [S2]<br>Amide III [S2]
1237–1240	Proteins, lipids & DNA/RNA	Amide III and CH_2 wagging of glycine and proline [S2]<br>PO^{-2} antisymmetric stretch [S10]<br>Amide III, thymine, adenine and =CH bend of lipids [S3]
1300–1305	Proteins, lipids & DNA/RNA	CH_2 deformation in lipids, Adenine and Cytosine [S2]<br>Lipids, proteins (Amide III), Adenine and Cytosine [S6]<br>CH_2 twist in lipids [S3]<br>Peak indicative of phospholipids [S8]
1335 –1339	Proteins & DNA/RNA	Guanine and CH_3CH_2 wagging of collagen [S2]<br>Adenine, phenylalanine and CH deformation [S6]
1440–1447	Lipids & proteins	CH_2 deformation in lipids and proteins [S2, S6]<br>CH in carbohydrates [S12]
1450 –1485		C=N stretch [S2]<br>Guanine, adenine, CH deformation of proteins and lipids [S4]<br>Guanine, adenine, CH deformation of proteins, lipids and carbohydrates [S3]<br>Guanine, adenine, CH deformation of proteins, lipids and carbohydrates [S2, S3]
1548–1554	Proteins	Tryptophan [S2]<br>Amide III [S12]
1573–1578	DNA/RNA	Guanine and adenine [S2–S4]<br>Peak indicative of lipids [S8]
1600–1607	Proteins	C=C of phenylalanine and tyrosine [S3]<br>Amide I C=O stretch and phenylalanine [S2]
1651–1660	Lipids & proteins	Amide I and C=C stretch of lipids [S2–S4]

Continued on next page

TABLE S3 (continued): Biomolecular peak-assignments

**Peak Range (cm^{-1})**	**General assignment**	**Detailed biomolecular assignments**
		Amide I [S8, S11, S12]<br>C=C stetch of lipids [S6]
1680–1685	Lipids & proteins	Amide I [S2]<br>Amide I and C=C stretch of lipids [S3]
1729–1743	Lipids	C=O ester group in lipids [S2, S3]

D. PCA—confusion matrix table

Following PCA, PCA-LDA was performed, which was then cross-validated using a leave-one-out prediction algorithm. The overall prediction accuracy was 84%. The confusion matrix with the predicted accuracy of the PCA-LDA classifications for each hTERT MSC-line is shown below.

TABLE S4. Confusion matrix showing the predicted PCA-LDA classifications (18 PCs = 91% variance)

**MSC-line**	No. spectra (223)	Y101	Y201	Y102	Y202
Y101	111	105 (**94.6%**)	0 (0%)	6 (5.4%)	0 (0%)
Y201	36	0 (0%)	25 (**69.5%**)	3 (8.3%)	8 (22.2%)
Y102	40	1 (2.5%)	1 (2.5%)	28 (**70.0%**)	10 (25%)
Y202	36	0 (0%)	3 (8.3%)	4 (11.1%)	29 (**80.6%**)

E. KNN results

Tables showing the KNN-classification model results for each cell-type classified against the hTERT MSCs. Here, D specifies a "direct" PIR-match within the SE uncertainty and C a "closest" PIR-match outside of the SE uncertainty. $\%_{match}$(D) refers to the percentage of PIR markers in each panel that are directly matched.

TABLE S5. KNN results for CD317+MSCs. The $\%_{match}$(D) was assessed over all PIR markers in each panel.

**Panel**	PIR matches	$\%_{match}$(D)
932	618 (D). 639 (D). 755 (D). 824 (D). 875 (D). 971 (C). 999 (D). 1028 (D). 1060 (C). 1678 (D).	80%
971	618 (C). 716 (D). 849 (D). 875 (D). 1085 (D). 1124 (D). 1170 (D). 1305 (C). 1445(C). 1604 (D). 1678 (C). 1732 (D).	67%
1060	618 (C). 639 (C). 664 (D). 696(C). 716 (C). 742 (C). 755 (C). 779 (C). 824 (C). 849 (C). 875 (C). 891 (D). 932(C). 999 (C). 1028 (C). 1085 (C). 1100 (D). 1124 (C). 1152 (D). 1170 (C). 1203 (C). 1241 (C). 1305 (C). 1335 (C). 1445 (C). 1550 (D). 1573 (D). 1604 (C). 1654 (C). 1678 (C).	20%
1085	639 (C). 696 (C). 1060 (C). 1203 (D). 1550 (D). 971 (D). 999 (D). 1335 (D). 1654 (D).	67%
1445	696 (D). 742 (D). 971 (C). 1060 (C). 1335 (D). 1473 (C).	50%
1473	639 (D). 664 (C) 716 (C). 755 (D). 779 (C). 849 (C). 891 (D). 932 (D). 1028 (D). 1060 (C). 1085 (D). 1100 (C). 1124 (D). 1203 (D). 1241 (C). 1305 (C). 1335 (C). 1445(C). 1550 (C). 1573 (C). 1604 (D). 1654 (C). 1678 (D).	43%

TABLE S6. KNN results for HDFs. The $\%_{match}$(D) was assessed over all PIR markers in each panel.

**Panel**	PIR matches	$\%_{match}$(D)
932	618 (D). 639 (D). 755 (D). 824 (D). 875 (D). 971 (C). 999 (D). 1028 (D). 1060 (D). 1678 (D).	90%
971	618 (D). 716 (D). 849 (D). 875 (D). 1085 (D). 1124 (D). 1170 (D). 1305 (D). 1445(D). 1604 (D). 1678 (D). 1732 (C).	92%
1060	618 (C). 639 (D). 664 (C). 696(C). 716 (D). 742 (D). 755 (C). 779 (C). 824 (C). 849 (C). 875 (C). 891 (C). 932(D). 999 (C). 1028 (C). 1085 (D). 1100 (D). 1124 (C). 1152 (D). 1170 (D). 1203 (C). 1241 (D). 1305 (D). 1335 (C). 1445 (C). 1550 (C). 1573 (C). 1604 (D). 1654 (C). 1678 (D).	40%
1085	639 (D). 696 (D). 1060 (D). 1203 (D). 1550 (D). 971 (D). 999 (D). 1335 (D). 1654 (D).	100%
1445	696 (C). 742 (C). 971 (D). 1060 (C). 1335 (D). 1473 (C).	33%
1473	639 (D). 664 (D) 716 (D). 755 (C). 779 (D). 849 (C). 891 (C). 932 (D). 1028 (C). 1060 (D). 1085 (D). 1100 (D). 1124 (D). 1203 (C). 1241 (C). 1305 (C). 1335 (C). 1445(C). 1550 (C). 1573 (D). 1604 (D). 1654 (C). 1678 (D).	52%

TABLE S7. KNN results for K72 cell 1. The $\%_{match}$(D) was assessed over all PIR markers in each panel.

**Panel**	PIR matches	$\%_{match}$(D)
932	618 (C). 639 (D). 755 (D). 824 (C). 875 (D). 971 (C). 999 (D). 1028 (D). 1060 (D). 1678 (D).	70%
971	618 (C). 716 (D). 849 (D). 875 (D). 1085 (D). 1124 (D). 1170 (D). 1305 (D). 1445 (D). 1604 (D). 1678 (D). 1732 (C).	83%
1060	618 (C). 639 (D). 664 (C). 696(C). 716 (C). 742 (C). 755 (C). 779 (C). 824 (C). 849 (D). 875 (C). 891 (D). 932(D). 999 (C). 1028 (C). 1085 (D). 1100 (D). 1124 (D). 1152 (C). 1170 (D). 1203 (C). 1241 (D). 1305 (C). 1335 (D). 1445 (D). 1550 (C). 1573 (C). 1604 (D). 1654 (D). 1678 (C).	47%
1085	639 (D). 696 (D). 971 (D). 999 (D). 1060 (D). 1203 (D). 1335 (D). 1550 (D). 1654 (D).	100%
1445	696 (D). 742 (C). 971 (D). 1060 (D). 1335 (D). 1473 (D).	83%
1473	639 (D). 664 (D) 716 (D). 755 (D). 779 (D). 849 (C). 891 (D). 932 (D). 1028 (D). 1060 (C). 1085 (D). 1100 (C). 1124 (D). 1203 (D). 1241 (C). 1305 (D). 1335 (D). 1445(D). 1550 (C). 1573 (D). 1604 (D). 1654 (D). 1678 (D).	78%

TABLE S8. KNN results for K72 cell 2. The $\%_{match}$(D) was assessed over all PIR markers in each panel.

**Panel**	PIR matches	% match
932	618 (D). 639 (D). 755 (C). 824 (C). 875 (C). 971 (D). 999 (C). 1028 (C). 1060 (D). 1678 (C).	40%
971	618 (D). 716 (C). 849 (C). 875 (C). 1085 (C). 1124 (C). 1170 (D). 1305 (C). 1445(C). 1604 (C). 1678 (C). 1732 (C).	17%
1060	618 (C). 639 (C). 664 (C). 696(D). 716 (D). 742 (C). 755 (D). 779 (D). 824 (C). 849 (D). 875 (D). 891 (C). 932(D). 999 (C). 1028 (C). 1085 (D). 1100 (D). 1124 (D). 1152 (D). 1170 (C). 1203 (D). 1241 (D). 1305 (D). 1335 (D). 1445 (D). 1550 (D). 1573 (D). 1604 (D). 1654 (D). 1678 (D).	70%
1085	639 (C). 696 (D). 971 (C). 999 (D). 1060 (C). 1203 (D). 1335 (D). 1550 (C). 1654 (D).	67%
1445	696 (D). 742 (C). 971 (C). 1060 (D). 1335 (D). 1473 (D).	67%
1473	639 (D). 664 (D) 716 (C). 755 (D). 779 (D). 849 (D). 891 (D). 932 (D). 1028 (D). 1060 (C). 1085 (D). 1100 (C). 1124 (D). 1203 (D). 1241 (D). 1305 (D). 1335 (D). 1445(D). 1550 (C). 1573 (D). 1604 (D). 1654 (D). 1678 (D).	83%

TABLE S9. KNN results for K72 cell 3. The $\%_{match}$(D) was assessed over all PIR markers in each panel.

**Panel**	PIR matches	% match
932	618 (D). 639 (D). 755 (C). 824 (D). 875 (D). 971 (C). 999 (D). 1028 (D). 1060 (D). 1678 (D)	80%
971	618 (D). 716 (C). 849 (C). 875 (C). 1085 (C). 1124 (C). 1170 (C). 1305 (C). 1445(C). 1604 (C). 1678 (C). 1732 (C).	8%
1060	618 (D). 639 (D). 664 (D). 696(D). 716 (C). 742 (D). 755 (C). 779 (C). 824 (D). 849 (C). 875 (C). 891 (D). 932(D). 999 (D). 1028 (C). 1085 (D). 1100 (C). 1124 (C). 1152 (C). 1170 (C). 1203 (C). 1241 (C). 1305 (C). 1335 (C). 1445 (C). 1550 (C). 1573 (D). 1604 (D). 1654 (C). 1678 (D).	43%
1085	639 (C). 696 (D). 971 (C). 999 (D). 1060 (D). 1203 (D). 1335 (D). 1550 (D). 1654 (D).	78%
1445	696 (D). 742 (D). 971 (C). 1060 (C). 1335 (D). 1473 (D).	67%
1473	639 (D). 664 (D) 716 (C). 755 (C). 779 (D). 849 (D). 891 (D). 932 (D). 1028 (D). 1060 (C). 1085 (D). 1100 (C). 1124 (D). 1203 (D). 1241 (D). 1305 (D). 1335 (D). 1445(D). 1550 (C). 1573 (D). 1604 (D). 1654 (D). 1678 (D).	78%

F. Table summaries of PIR-specific, percentage standard error (%SE) decay rates.

The %SE decay rates (tau / τ) are obtained from the fitted convergence plots (Figs. S15–S20).

TABLE S10. 971/1085 %SE convergence results Y101 hTERT MSC-line.

Cell line	Passage (p)	Decay rate (τ)
Y101	64	10.0 ± 0.9
Y101	65	9.0 ± 0.9
Y101	66	7 ± 0.8
Y101	85	12 ± 0.7
Y101	91	13 ± 1
Y101	93	9 ± 1
**Y101**	Ave.	**10.0 (6.2–14)**

TABLE S11. 971/1085 %SE convergence results Y201 hTERT MSC-line

Cell line	Passage (p)	Decay rate (τ)
Y201	60	11.0 ± 0.6
Y201	63	6.0 ± 0.3
**Y201**	**Ave.**	**8.5 (5.7–11.6)**

TABLE S12. 971/1085 %SE convergence results Y102 hTERT MSC-line

Cell line	Passage (p)	Decay rate (τ)
Y102	66	11 ± 1
Y102	79	6.0 ± 0.5
**Y102**	**Ave.**	**8.5 (5.5–12)**

TABLE S13. 971/1085 %SE convergence results Y202 hTERT MSC-line

Cell line	Passage (p)	Decay rate (τ)
Y202	76	12.0 ±0.7
Y202	80	11 ±1
**Y202**	**Ave.**	**11.5 (10.0–12.7)**

TABLE S14. 1654/1085 %SE convergence results Y101 hTERT MSC-line

Cell line	Passage	Decay rate (τ)
Y101	64	9.0 ± 0.8
Y101	65	8.0 ± 0.7
Y101	66	5.0 ± 0.1
Y101	85	11.0 ± 1
Y101	91	8 ± 1
Y101	93	5 ± 1
**Y101**	Ave.	**7.7 (4–12)**

TABLE S15. 1654/1085 %SE convergence results Y201 hTERT MSC-line

Cell line	Passage (p)	Decay rate (τ)
Y201	60	4.0 ± 0.6
Y201	63	3.0 ± 0.8
**Y201**	**Ave.**	**3.5 (2.2–4.6)**

TABLE S16. 1654/1085 %SE convergence results Y102 hTERT MSC-line

Cell line	Passage (p)	Decay rate (τ)
Y102	66	7 ± 1
Y102	79	8 ± 1
**Y102**	**Ave.**	**7.5 (6–9)**

TABLE S17. 1654/1085 %SE convergence results Y202 hTERT MSC-line

Cell line	Passage (p)	Decay rate (τ)
Y202	76	6 ±2
Y202	80	7.0 ±0.7
**Y202**	**Ave.**	**6.5 (4–8.0)**

II. SUPPLEMENTARY FIGURES

A. Convergence of statistical quantities

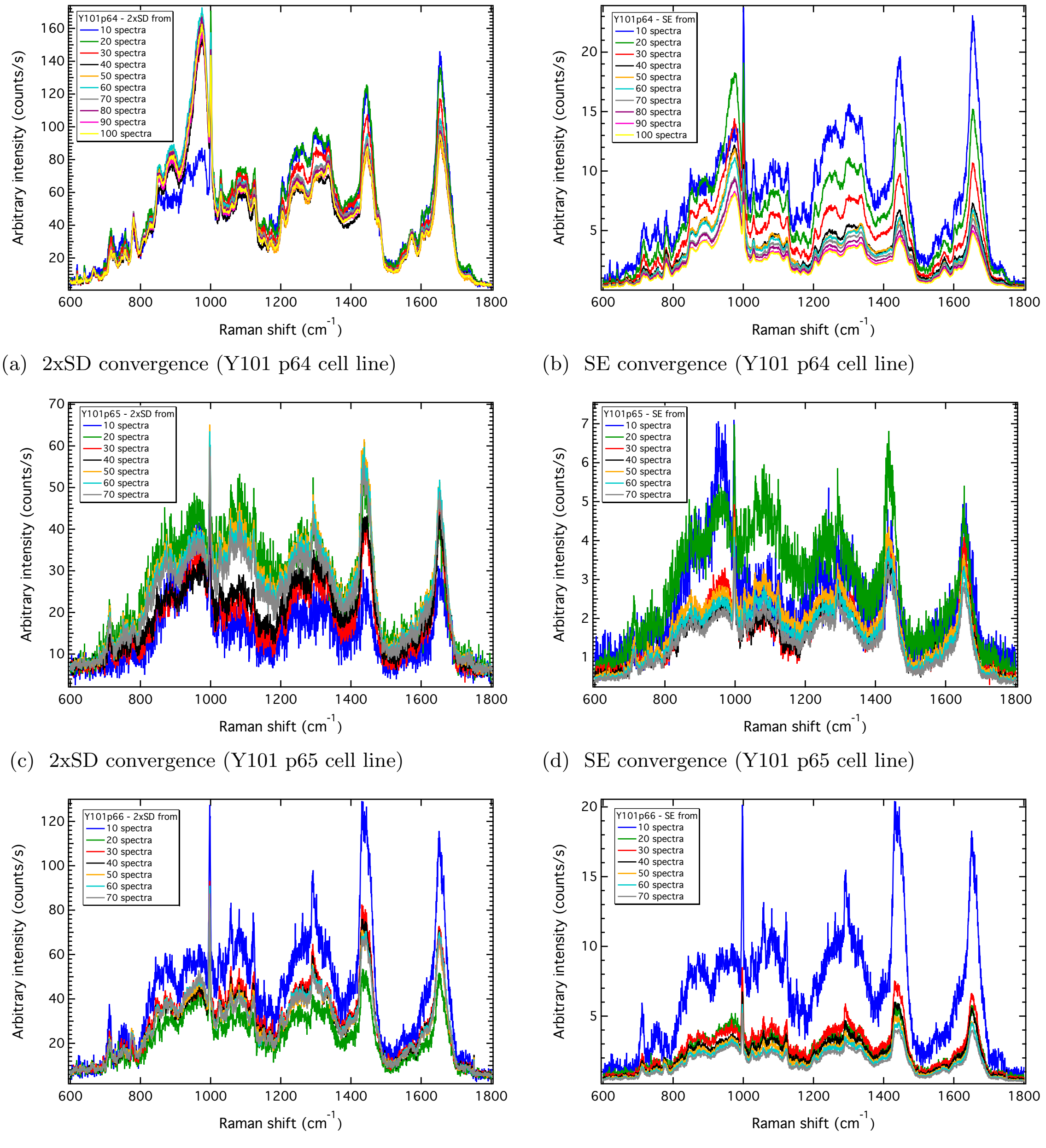

(a) 2xSD convergence (Y101 p64 cell line)

(b) SE convergence (Y101 p64 cell line)

(c) 2xSD convergence (Y101 p65 cell line)

(d) SE convergence (Y101 p65 cell line)

(e) 2xSD convergence (Y101 p66 cell line)

(f) SE convergence (Y101 p66 cell line)

FIG. S1. Convergence of twice the standard deviation (2xSD) and standard error of the mean (SE) as a function of the increasing number of spectra per spectral average for the Y101 p64, Y101 p65 and Y101 p66 hTERT MSC-lines (population-level). Here, p refers to the passage.

Convergence of statistical quantities, cont'd.

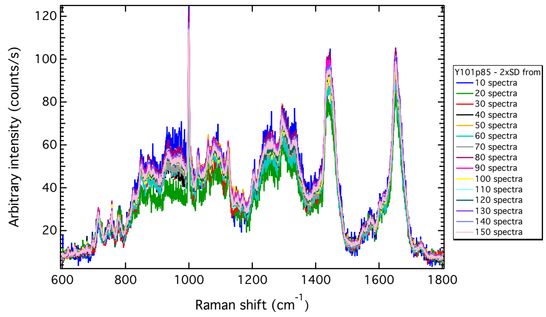

(a) 2xSD convergence (Y101 p85 cell line)

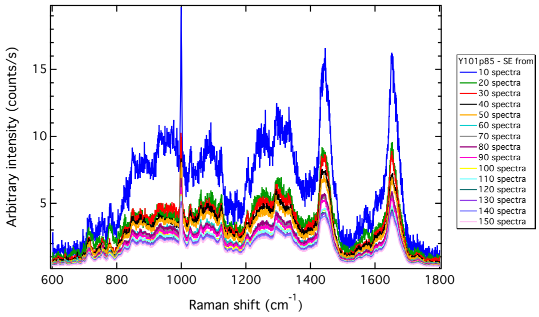

(b) SE convergence (Y101 p85 cell line)

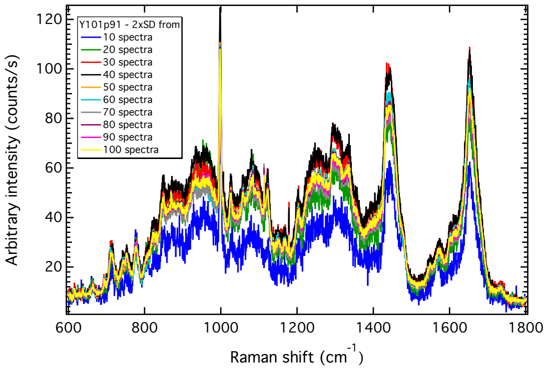

(c) 2xSD convergence (Y101 p91 cell line)

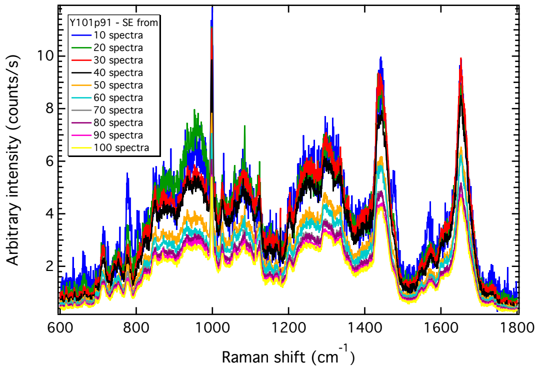

(d) SE convergence (Y101 p91 cell line)

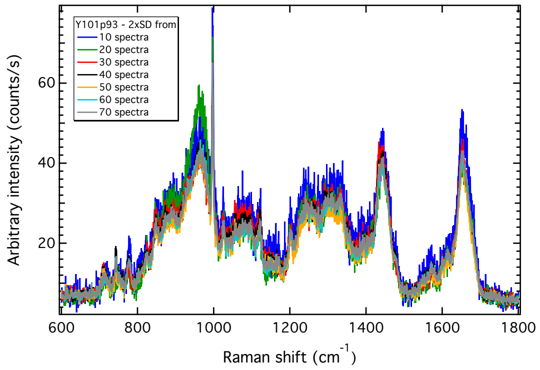

(e) 2xSD convergence (Y101 p93 cell line)

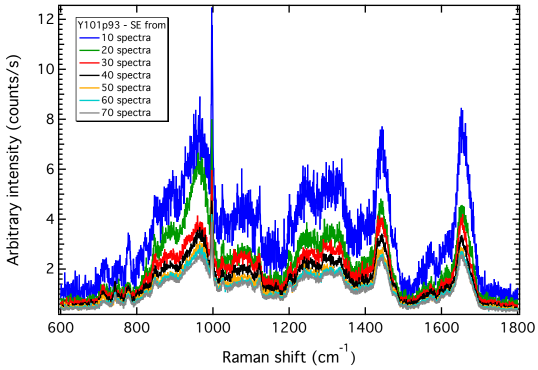

(f) SE convergence (Y101 p93 cell line)

FIG. S2. Convergence of twice the standard deviation (2xSD) and standard error of the mean (SE) as a function of the increasing number of spectra per spectral average for the Y101 p85, Y101 p91 and Y101 p93 hTERT MSC-lines (population level). Here, p refers to the passage.

Convergence of statistical quantities, cont'd.

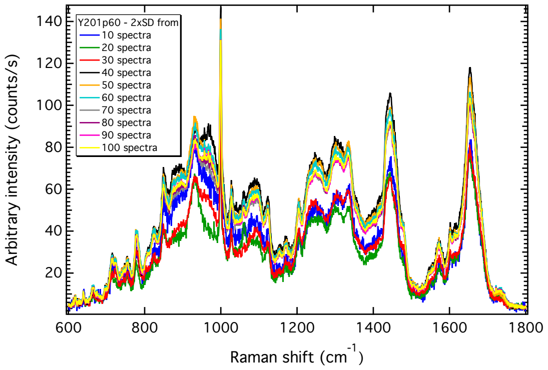

(a) 2xSD convergence (Y201 p60 cell line)

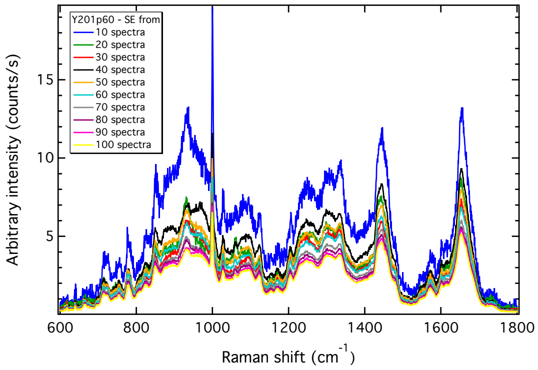

(b) SE convergence (Y201 p60 cell line)

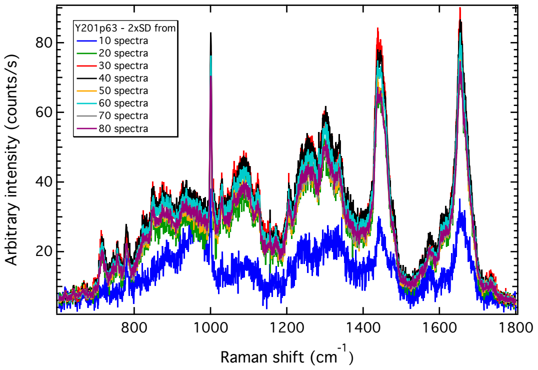

(c) 2xSD convergence (Y201 p63 cell line)

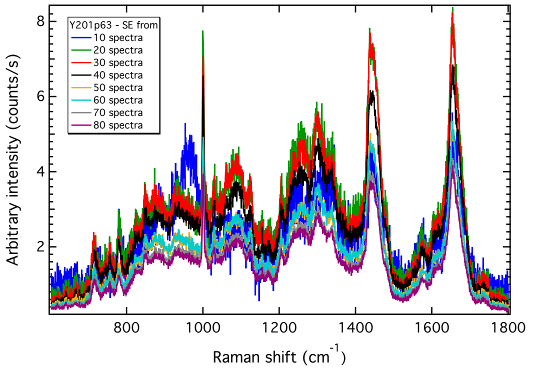

(d) SE convergence (Y201 p63 cell line)

FIG. S3. Convergence of twice the standard deviation (2xSD) and standard error of the mean (SE) as a function of the increasing number of spectra per spectral average for the Y201 p60 and Y201 p63 hTERT MSC-lines (population level). Here, p refers to the passage.

Convergence of statistical quantities, cont'd.

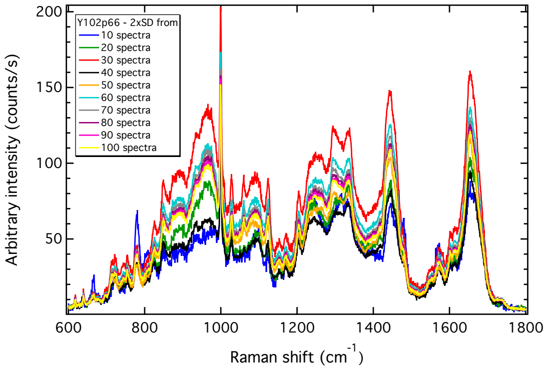

(a) 2xSD convergence (Y102 p66 cell line)

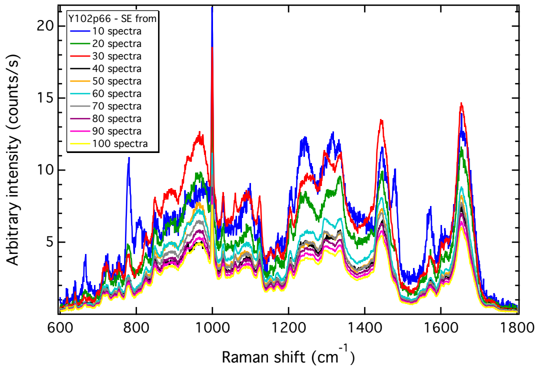

(b) SE convergence (Y102 p66 cell line)

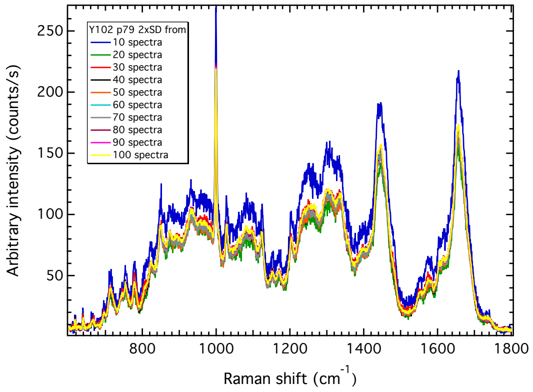

(c) 2xSD convergence (Y102 p79 cell line)

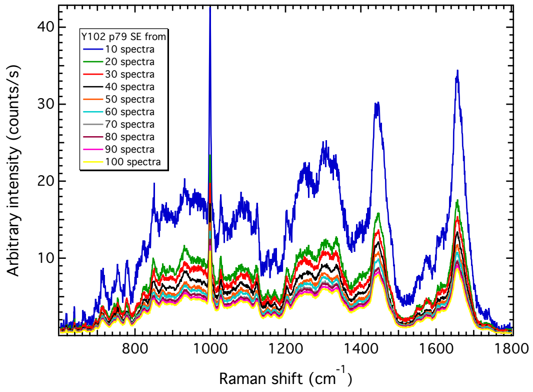

(d) SE convergence (Y102 p79 cell line)

FIG. S4. Convergence of twice the standard deviation (2xSD) and standard error of the mean (SE) as a function of the increasing number of spectra per spectral average for the Y102 p66 and Y102 p79 hTERT MSC-lines (population level). Here, p refers to the passage.

Convergence of statistical quantities, cont'd.

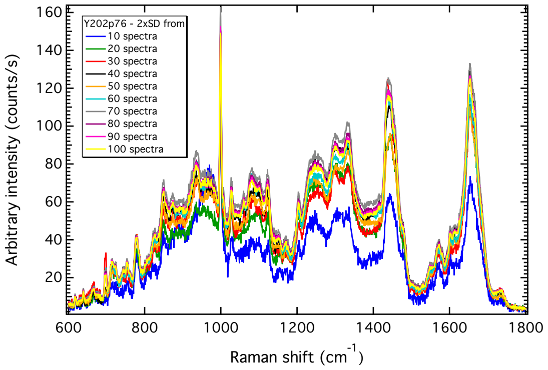

(a) 2xSD convergence (Y202 p76 cell line)

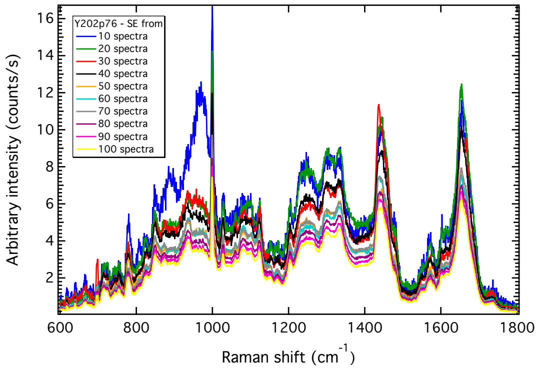

(b) SE convergence (Y202 p76 cell line)

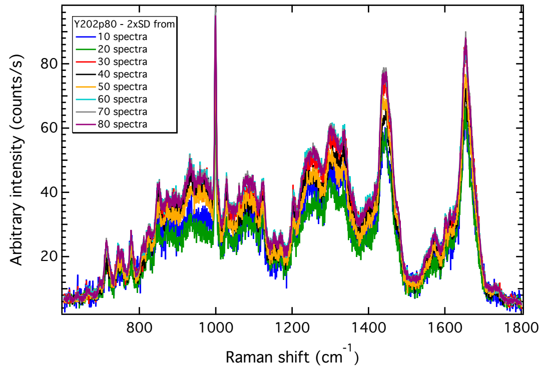

(c) 2xSD convergence (Y202 p80 cell line)

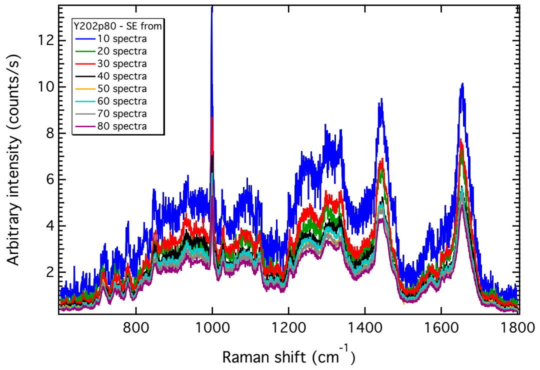

(d) SE convergence (Y202 p80 cell line)

FIG. S5. Convergence of twice the standard deviation (2xSD) and standard error of the mean (SE) as a function of the increasing number of spectra per spectral average for the Y202 p76 and Y202 p80 hTERT MSC-lines (population level). Here, p refers to the passage.

Convergence of statistical quantities, cont'd.

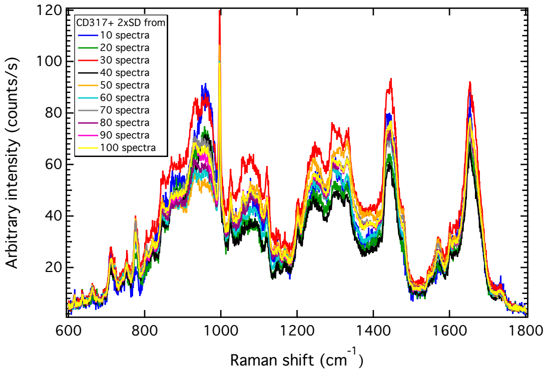

(a) 2xSD convergence (CD317+MSCs)

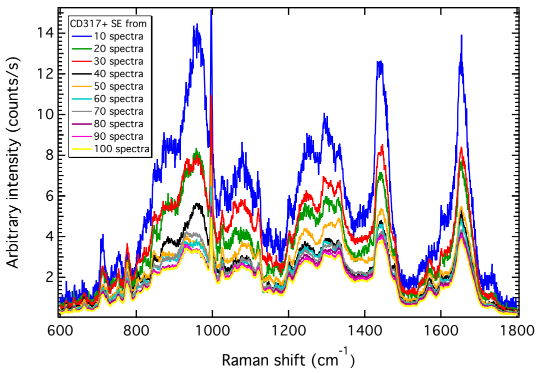

(b) SE convergence (CD317+MSCs)

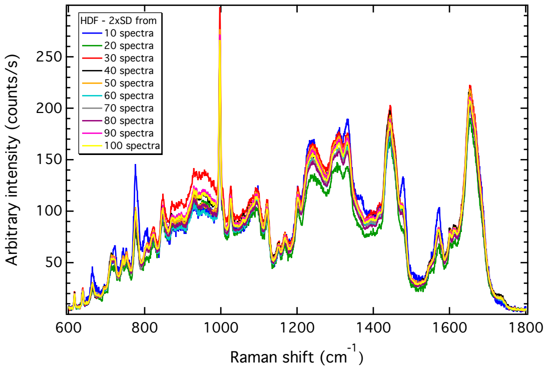

(c) 2xSD convergence (HDFs)

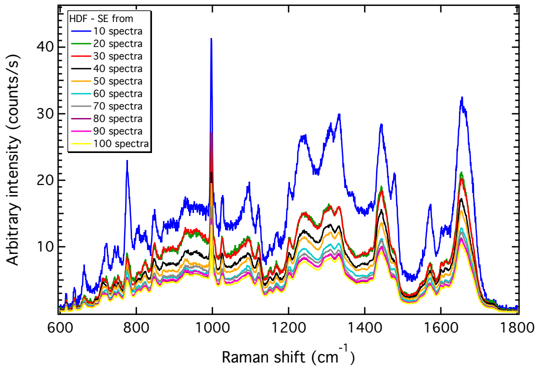

(d) SE convergence (HDFs)

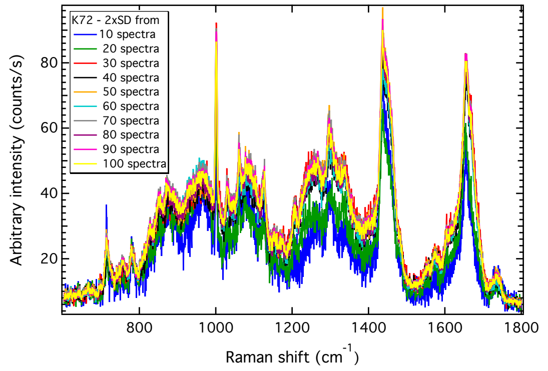

(e) 2xSD convergence (K72 primaries)

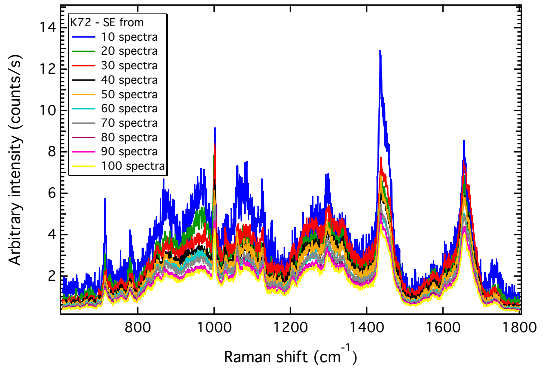

(f) SE convergence (K72 primaries)

FIG. S6. Convergence of twice the standard deviation (2xSD) and standard error of the mean (SE) as a function of the increasing number of spectra per spectral average for the CD317+MSC, HDF and K72 primaries (population level).

Convergence of statistical quantities, cont'd.

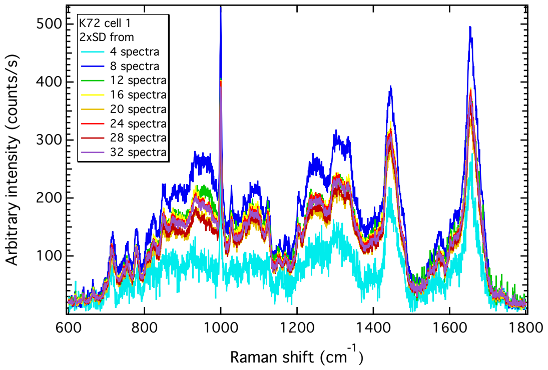

(a) 2xSD convergence (K72 cell 1)

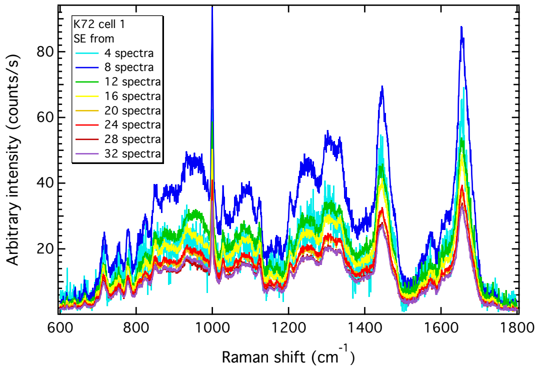

(b) SE convergence (K72 cell 1)

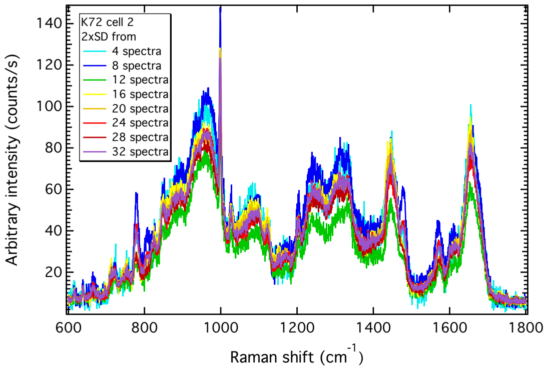

(c) 2xSD convergence (K72 cell 2)

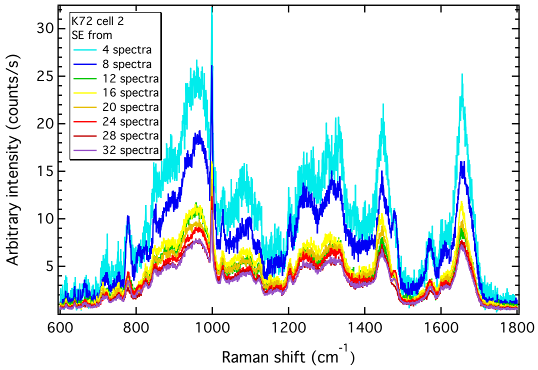

(d) SE convergence (K72 cell 2)

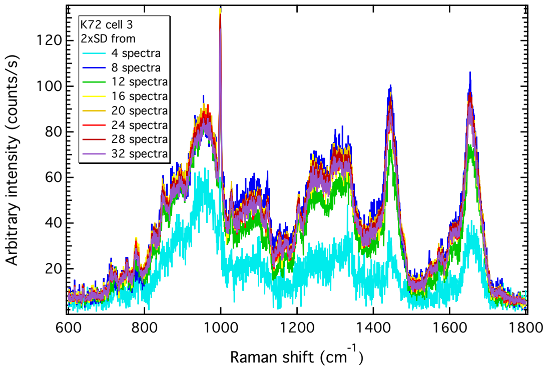

(e) 2xSD convergence (K72 cell 3)

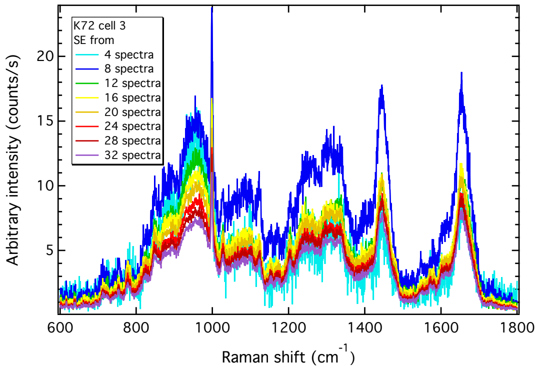

(f) SE convergence (K72 cell 3)

FIG. S7. Convergence of twice the standard deviation (2xSD) and standard error of the mean (SE) as a function of the increasing number of spectra per spectral average for single-cell, K72 primary MSCs 1, 2 and 3 (Raman maps).

B. PCA

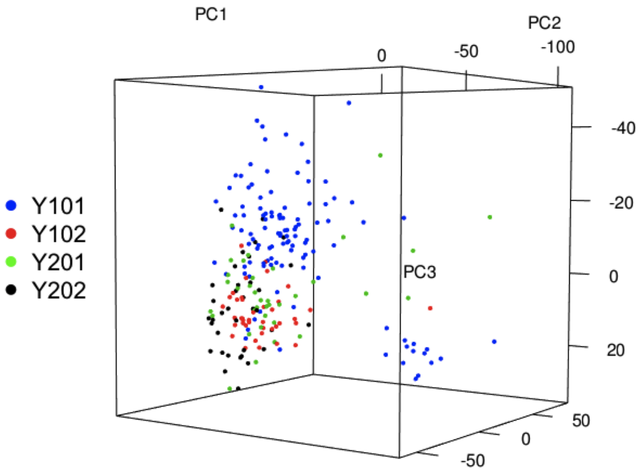

FIG. S8. PCA scatterplot for the hTERT MSCs (PC1 x PC2 x PC3) showing that PCA is not able to fully discriminate the four cell-lines, although the Y101 hTERT MSC-line shows near separation. Each point in the PCA plot is obtained from the spectral average of five spectra/cell nucleus. The first three PCs capture 66% of the total variance in the dataset, with PC1, PC2 and PC3 accounting for 32%, 22% and 12% of the total variance, respectively.

C. K72 primary MSC studies

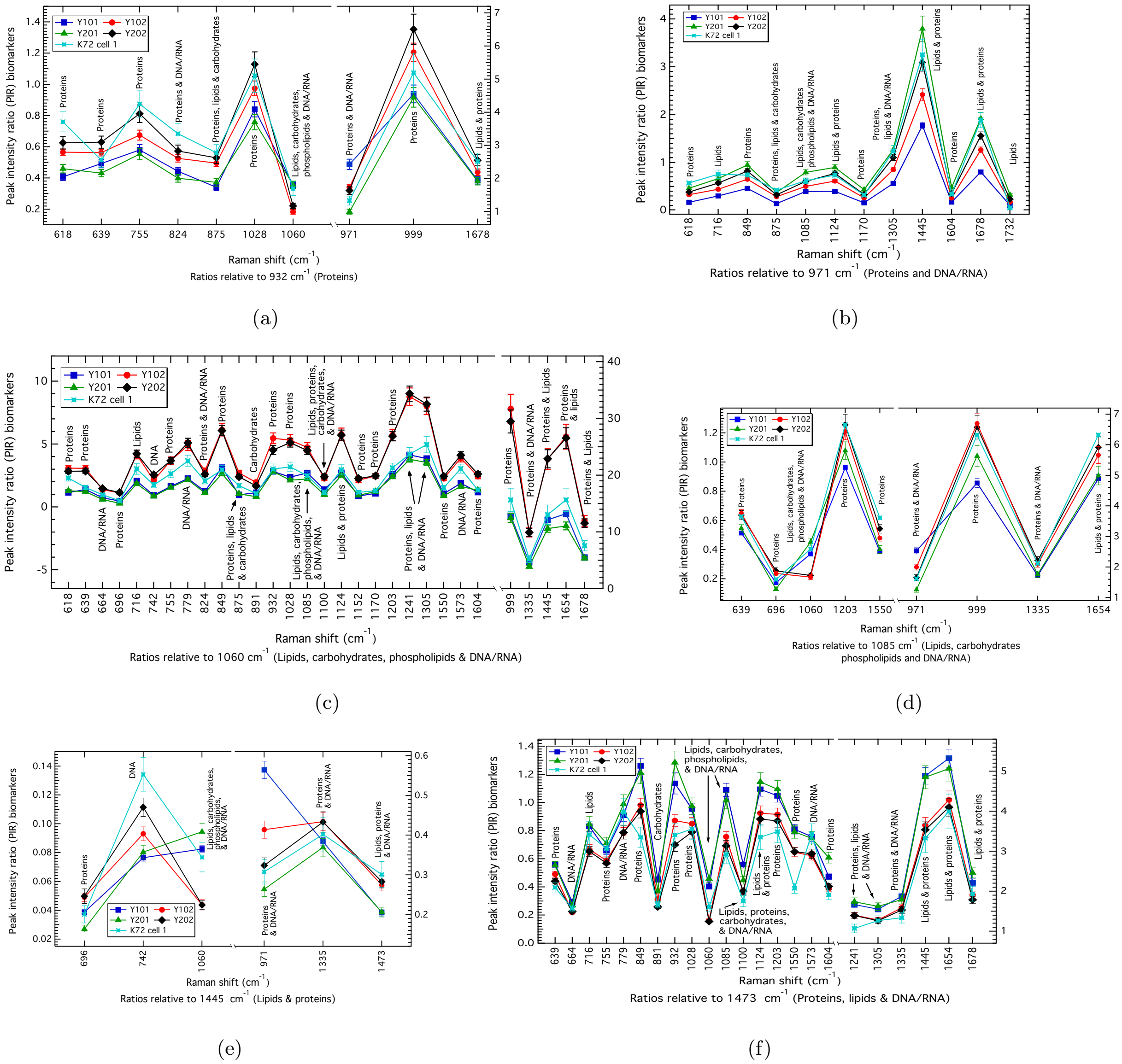

FIG. S9. PIR comparisons for the K72 primary cell 1 against the hTERT MSC biomarker panels. The uncertainties are the propagated standard error of the mean (SE) values associated with each PIR measurement.

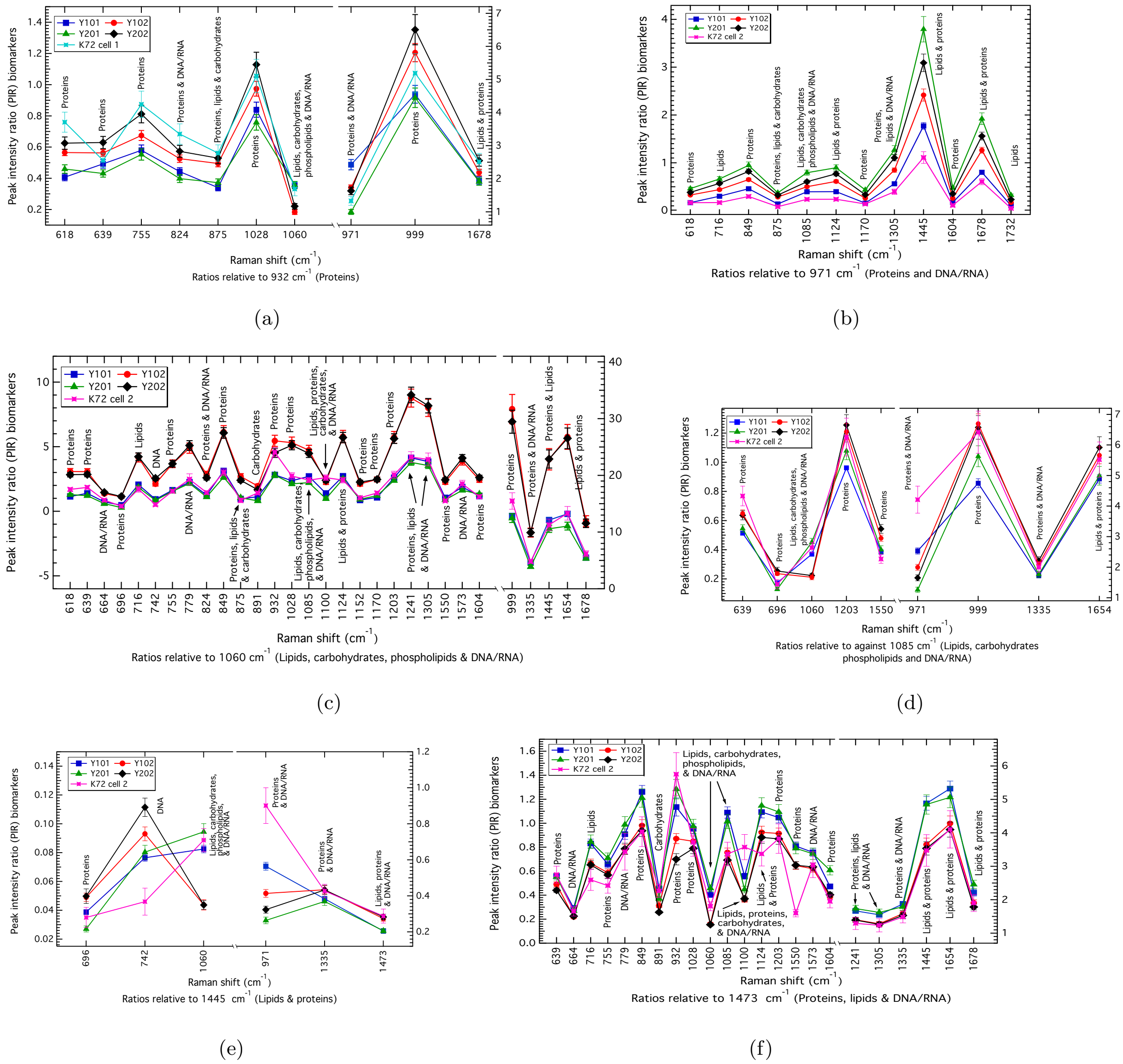

FIG. S10. PIR comparisons for the K72 primary cell 2 against the hTERT MSC biomarker panels. The uncertainties are the propagated standard error of the mean (SE) values associated with each PIR measurement.

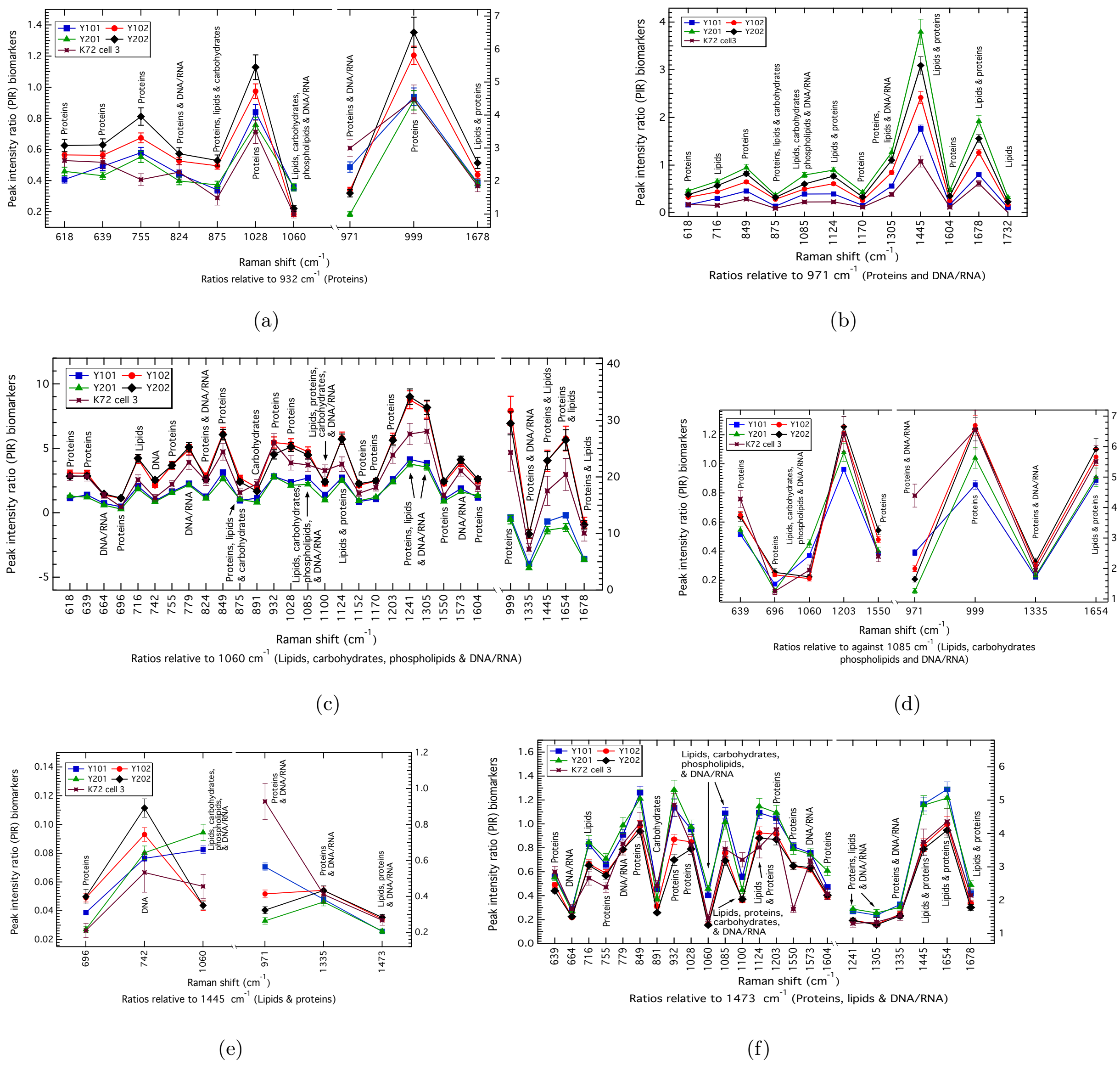

FIG. S11. PIR comparisons for the K72 primary cell 3 against the hTERT MSC biomarker panels. The uncertainties are the propagated standard error of the mean (SE) values associated with each PIR measurement.

D. Reproducibility tests

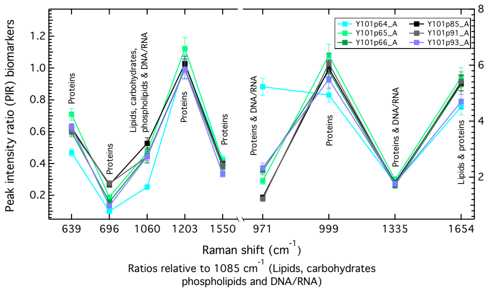

(a)

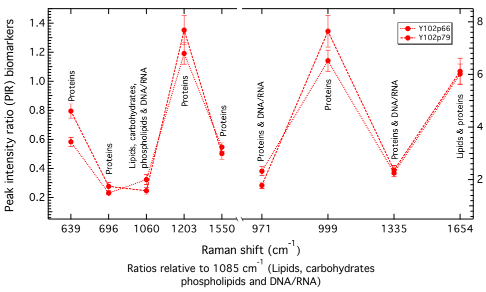

(b)

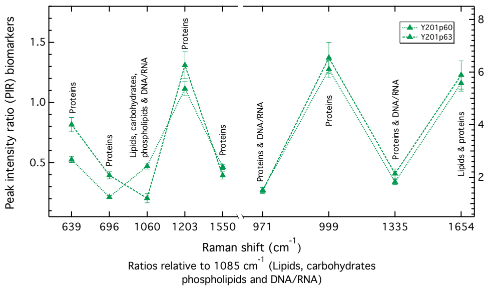

(c)

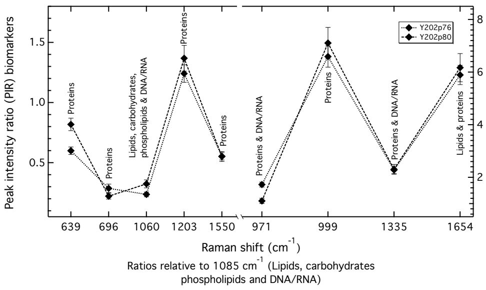

(d)

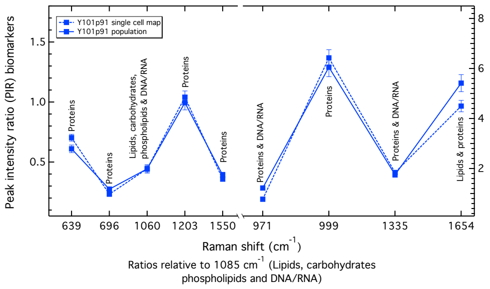

(e)

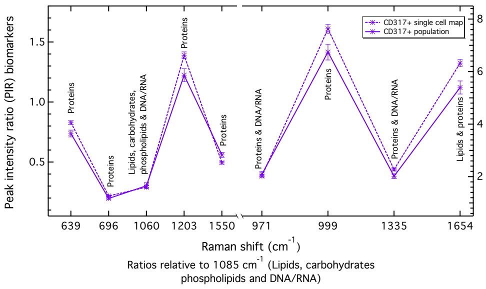

(f)

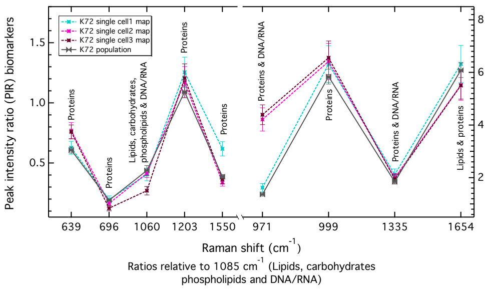

(g)

FIG. S12. Reproducibility tests for the 1085 cm^{-1} PIR panels between experimental replicates for the (a) Y101, (b) Y102, (c) Y201 and (d) Y202 MSC-lines showing different passages, p, and (e), (f) and (g) showing population and single cell comparisons for the Y101 p93 hTERT MSC-line, CD317+MSC and the K72 MSC primaries, respectively. Error bars are the propagated standard errors.

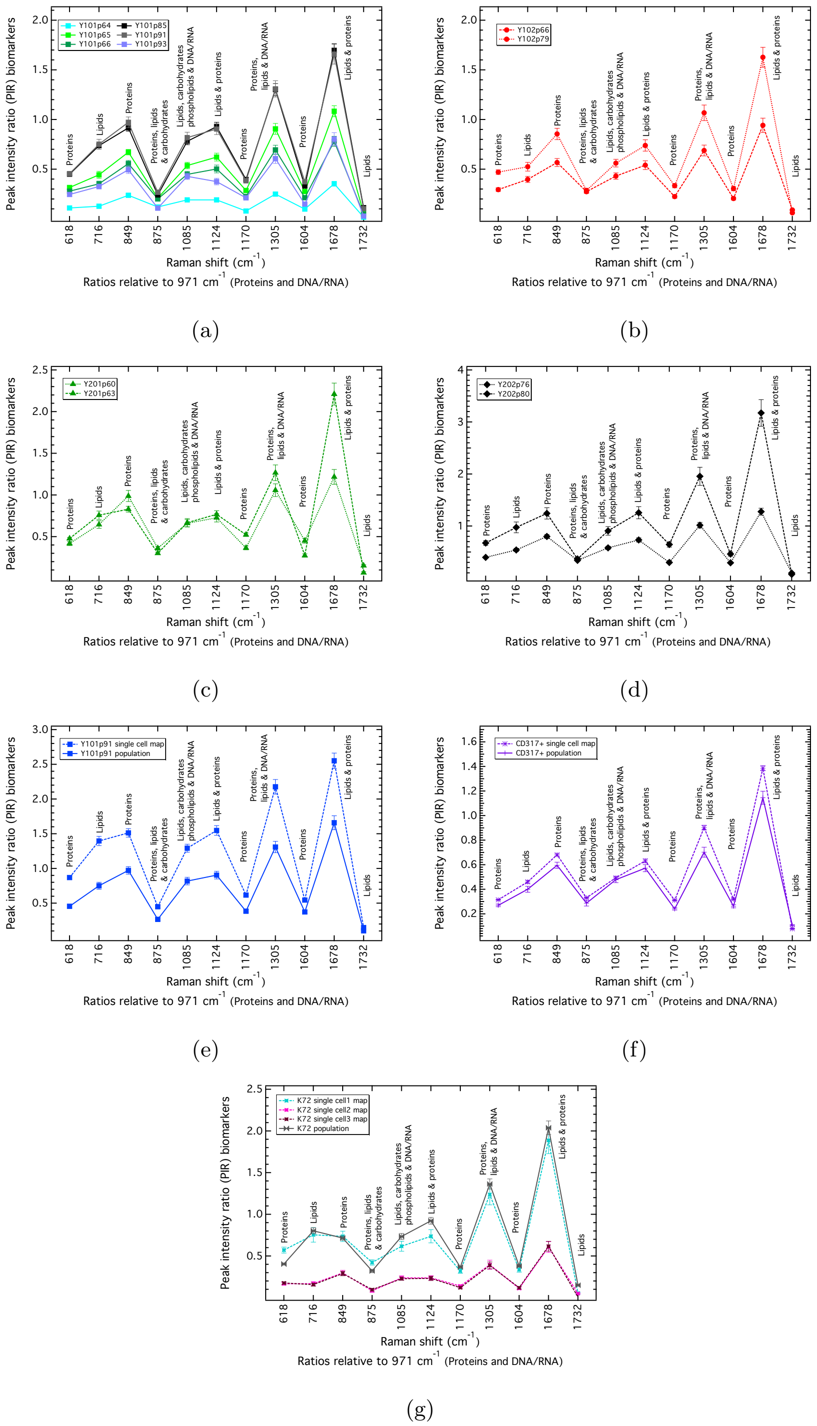

FIG. S13. Reproducibility tests for the 971 cm^{-1} PIR panels between experimental replicates for the (a) Y101, (b) Y102, (c) Y201 and (d) Y202 MSC-lines showing different passages, p, and (e), (f) and (g) showing population and single cell comparisons for the Y101 p93 hTERT MSC-line, CD317+MSC and the K72 MSC primaries, respectively. Error bars are the propagated standard errors.

E. DAPI experiments

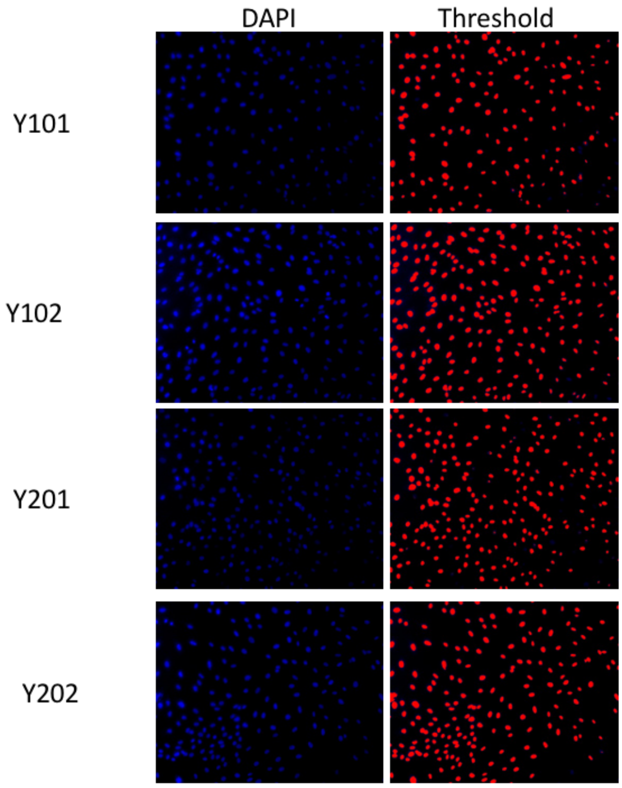

(a)

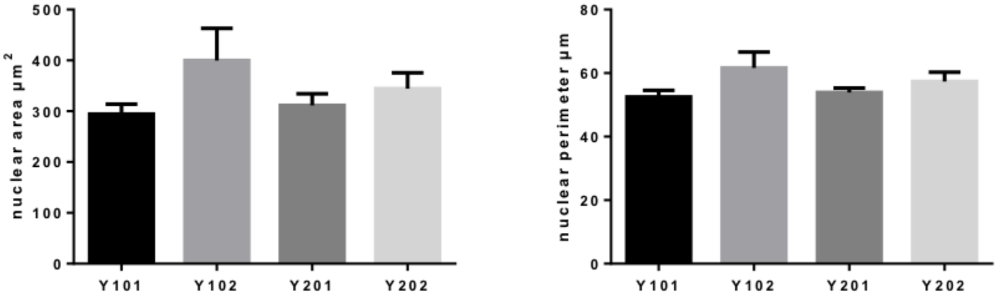

(b)

FIG. S14. (a) Fluorescent images and Image J thresholded counterparts of DAPI counter-stained hTERT MSC-lines. The figure shows a representative image of DAPI counter-stained nuclei of each hTERT MSC-line alongside its Image J thresholded counterpart. The Image J figure selects each object that it considers to be nuclei and gives it a number. Images were screened for nucleus validity: partial-objects or objects that had been incorrectly discriminated as nuclei were excluded from area and perimeter analyses. (b) Comparisons of the area and perimeter of the nuclei of hTERT MSC-lines. Nucleus area and perimeter of the four cell-lines were calculated using Image J processing of fluorescent images of cells counter-stained with DAPI. The average data $\pm$ standard deviation from > 67 cells are displayed.

F. PIR convergence tests

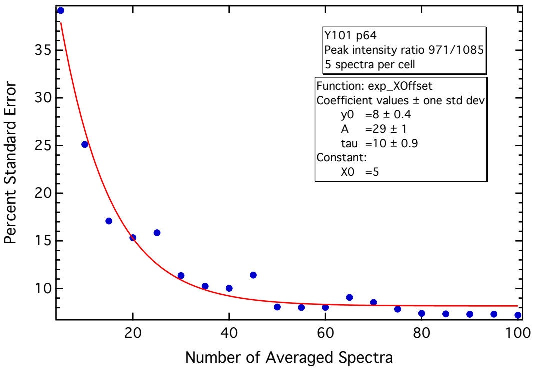

(a) Y101 p64

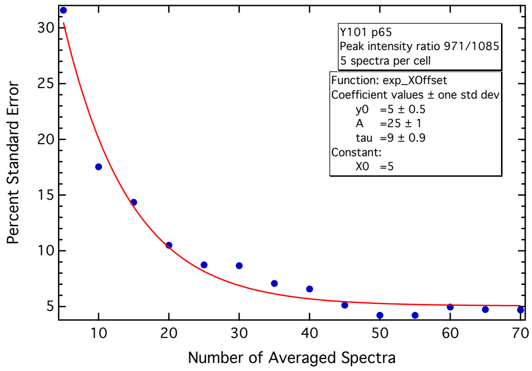

(b) Y101 p65

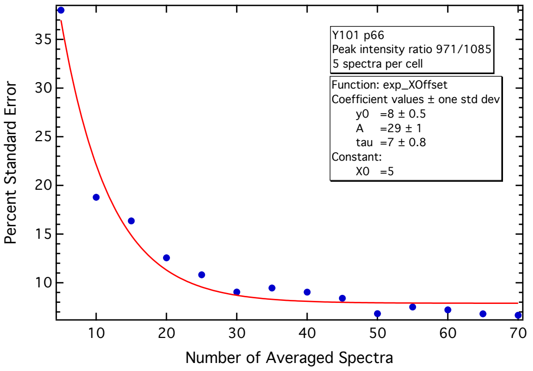

(c) Y101 p66

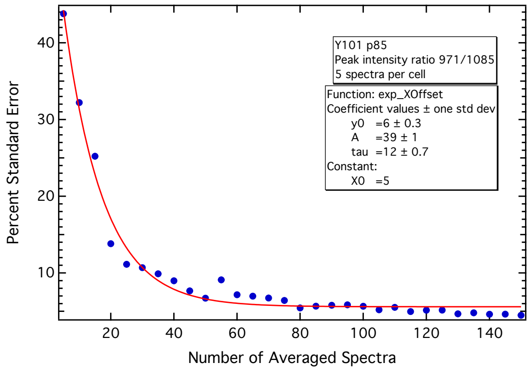

(d) Y101 p85

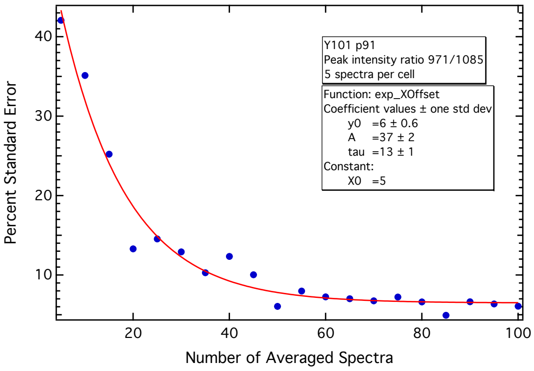

(e) Y101 p91

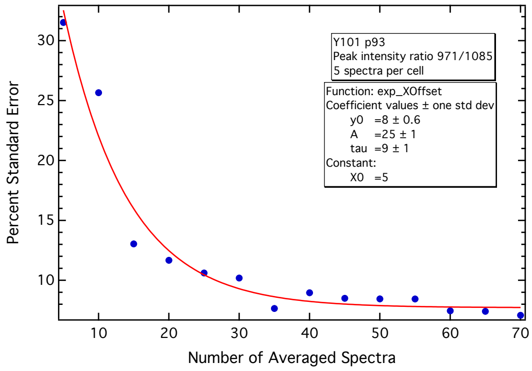

(f) Y101 p93

FIG. S15. 971/1085 PIR percentage standard error (%SE) convergence results as a function of the number of increasing spectra in the spectral average for the Y101 replicates. A decaying exponential function [$y = y_0 + A \exp\frac{x-x_0}{\tau}$] was used to fit the data. Each sub-figure shows the fitted values for the function parameters, where tau $= \tau$ denotes the decay constant. Here, p refers to the passage.

PIR convergence tests, cont'd.

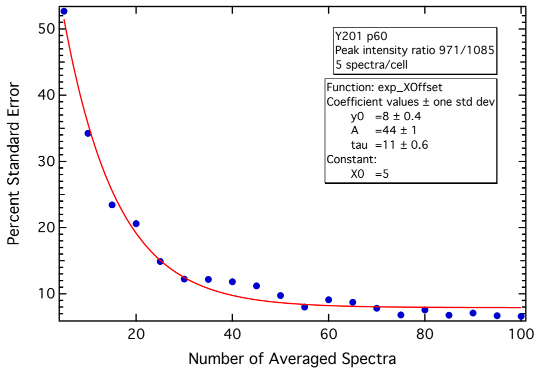

(a) Y201 p60

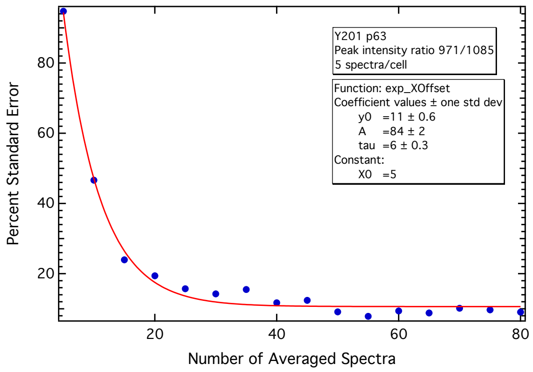

(b) Y201 p63

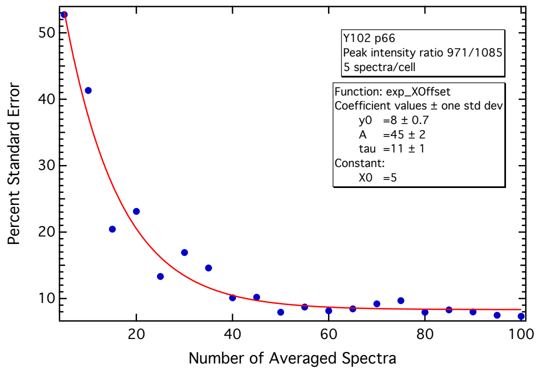

(c) Y102 p66

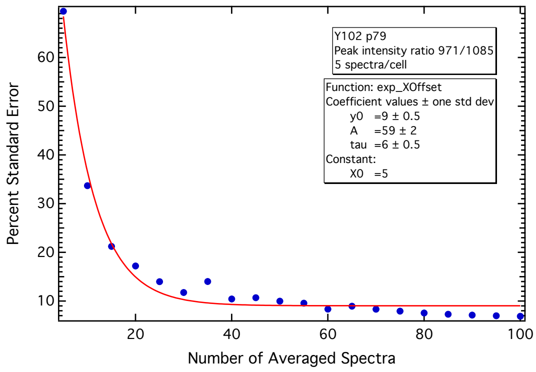

(d) Y102 p79

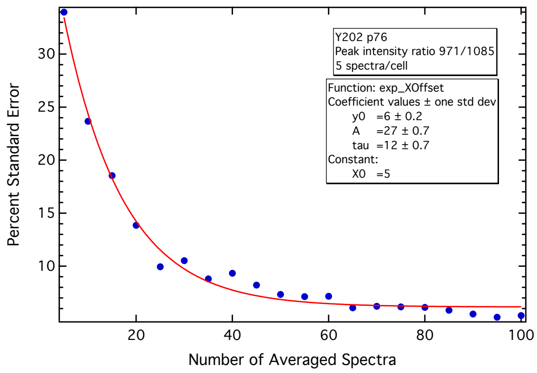

(e) Y202 p76

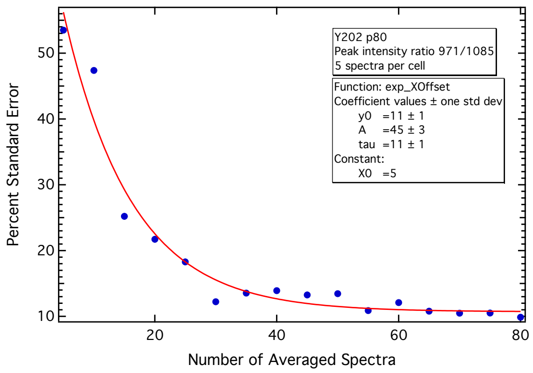

(f) Y202 p80

FIG. S16. 971/1085 PIR percentage standard error (%SE) convergence results as a function of the number of increasing spectra in the spectral average for the Y201, Y102 and Y202 replicates [Figs. (a) & (b), (c) & (d) and (e) & (f), respectively]. A decaying exponential function [$y = y_0 + A \exp \frac{x - x_0}{\tau}$] was used to fit the data. Each sub-figure shows the fitted values for the function parameters, where tau $= \tau$ denotes the decay constant. Here, p refers to the passage.

PIR convergence tests, cont'd.

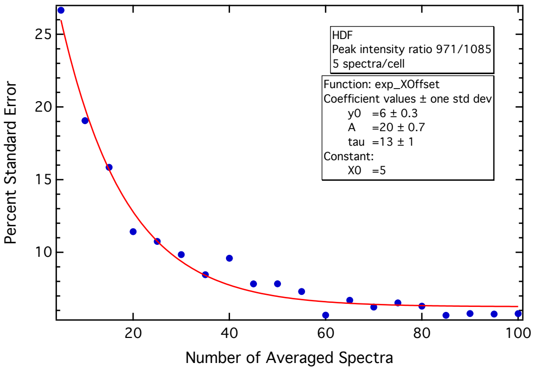

(a) HDFs

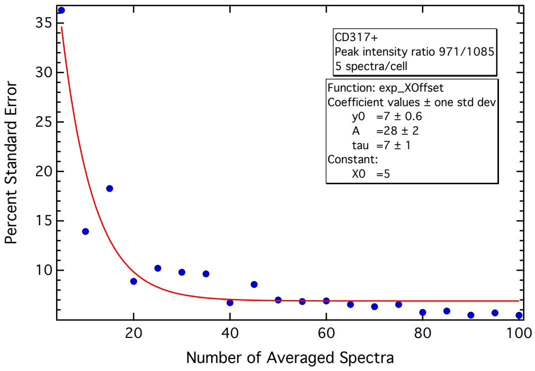

(b) CD317+

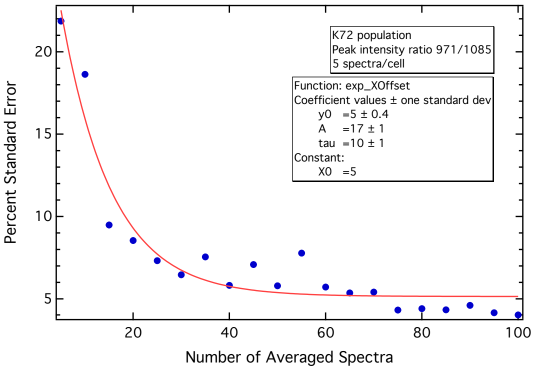

(c) K72–primary

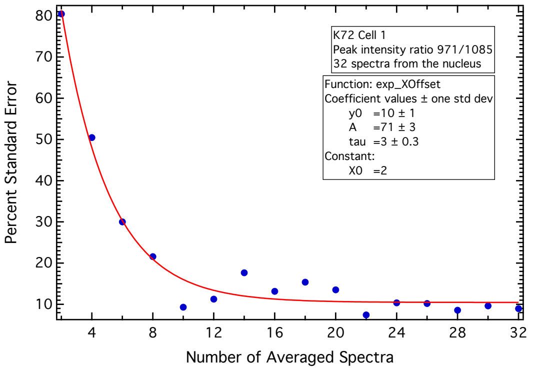

(d) K72 cell one

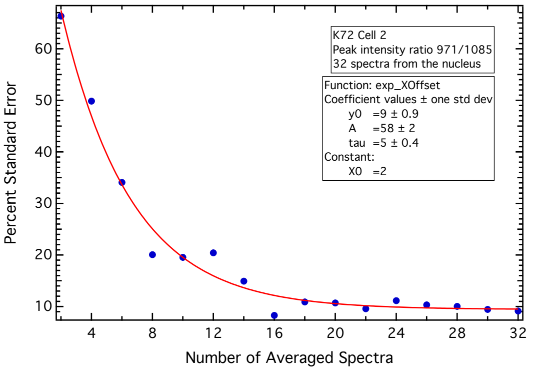

(e) K72 cell two

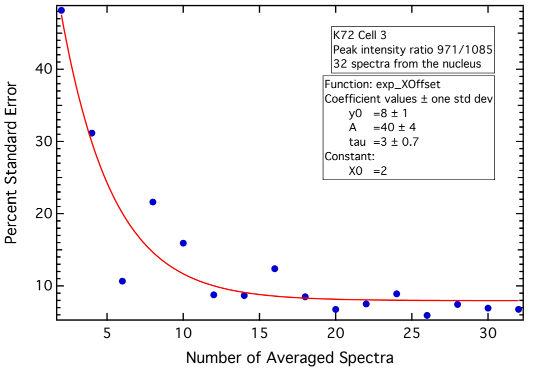

(f) K72 cell three

FIG. S17. 971/1085 PIR percentage standard error (%SE) convergence results as a function of the number of increasing spectra in the spectral average for the HDF, CD317+MSC and K72-primary cell-line populations [Figs. (a), (b) & (c), respectively]. A decaying exponential function [$y = y_0 + A \exp\frac{x - x_0}{\tau}$] was used to fit the data. Each sub-figure shows the fitted values for the function parameters, where tau = τ denotes the decay constant.

PIR convergence tests

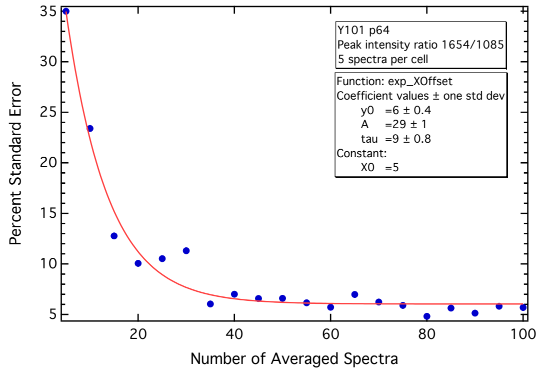

(a) Y101 p64

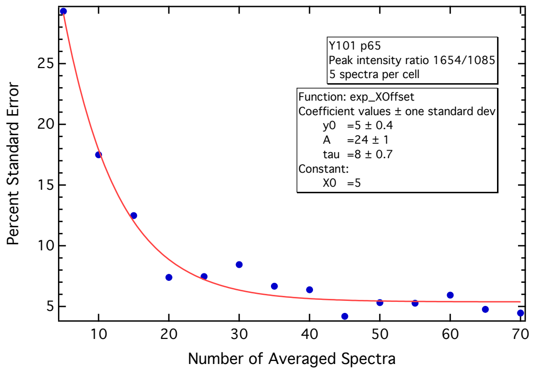

(b) Y101 p65

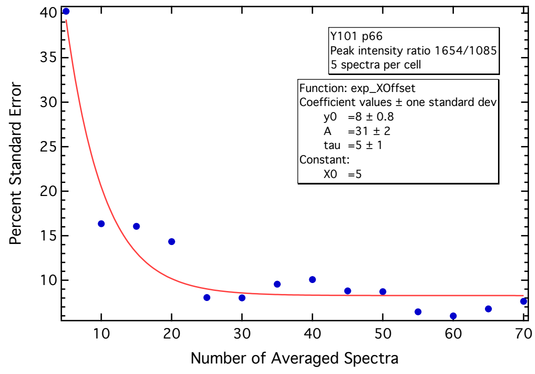

(c) Y101 p66

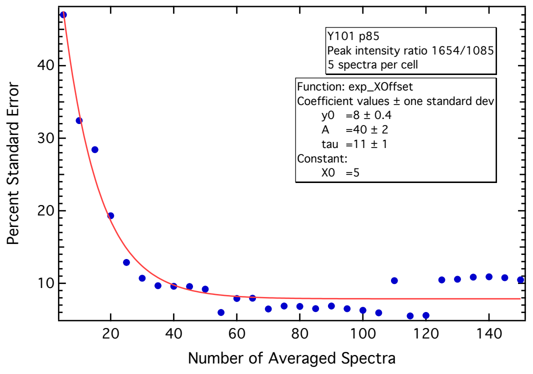

(d) Y101 p85

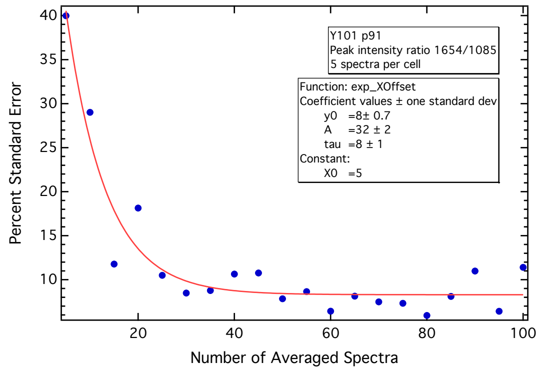

(e) Y101 p91

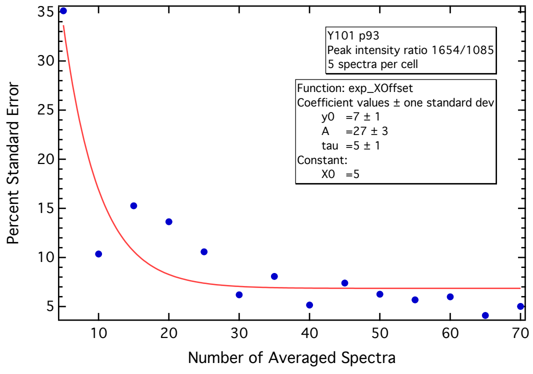

(f) Y101 p93

FIG. S18. 1654/1085 PIR percentage standard error (%SE) convergence results as a function of the number of increasing spectra in the spectral average for the Y101 replicates. A decaying exponential function $[y = y_0 + A \exp \frac{x - x_0}{\tau}]$ was used to fit the data. Each sub-figure shows the fitted values for the function parameters, where tau = τ denotes the decay constant. Here, p refers to the passage.

PIR convergence tests, cont'd.

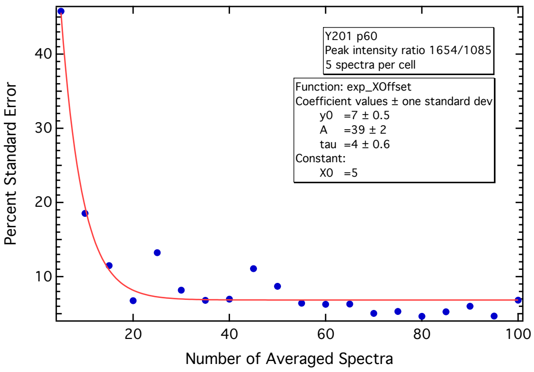

(a) Y201 p60

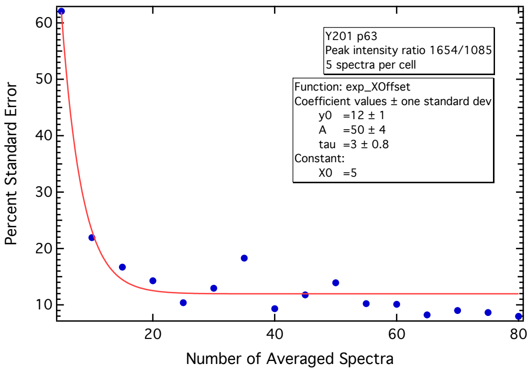

(b) Y201 p63

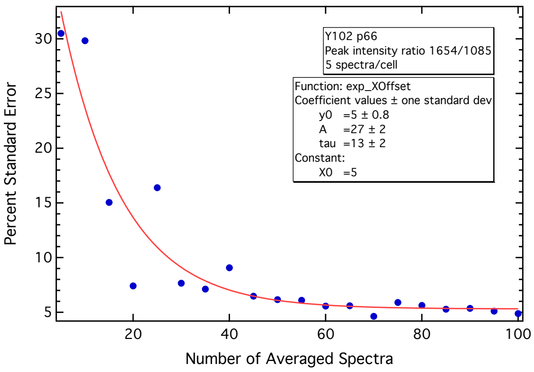

(c) Y102 p66

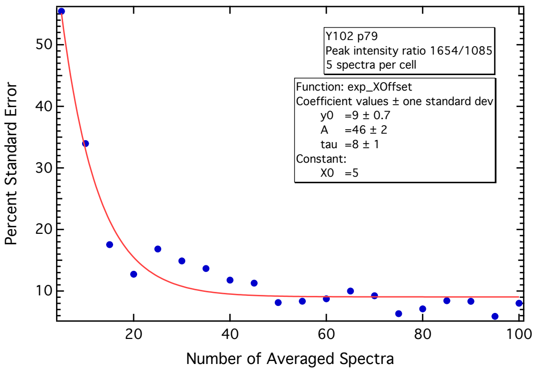

(d) Y102 p79

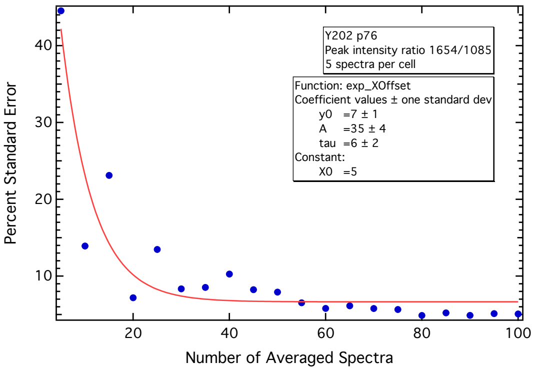

(e) Y202 p76

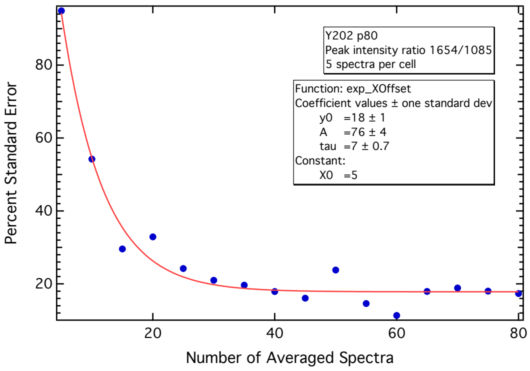

(f) Y202 p80

FIG. S19. 1654/1085 PIR percentage standard error (%SE) convergence results as a function of the number of increasing spectra in the spectral average for the Y201, Y102 and Y202 replicates [Figs. (a) & (b), (c) & (d) and (e) & (f), respectively]. A decaying exponential function [$y = y_0 + A \exp\frac{x-x_0}{\tau}$] was used to fit the data. Each sub-figure shows the fitted values for the function parameters, where tau = τ denotes the decay constant. Here, p refers to the passage.

PIR convergence tests, cont'd.

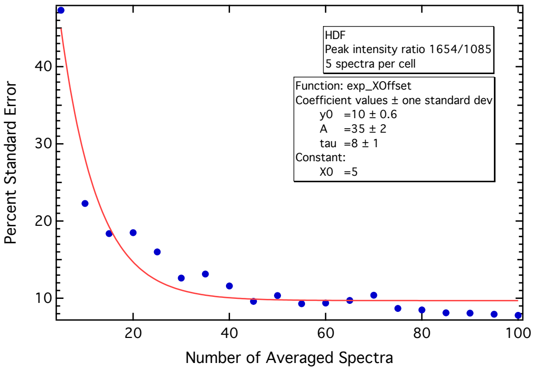

(a) HDFs

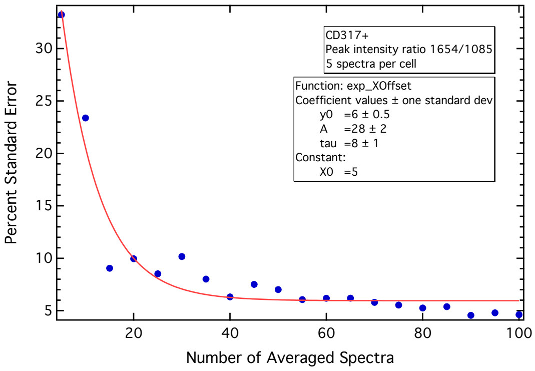

(b) CD317+

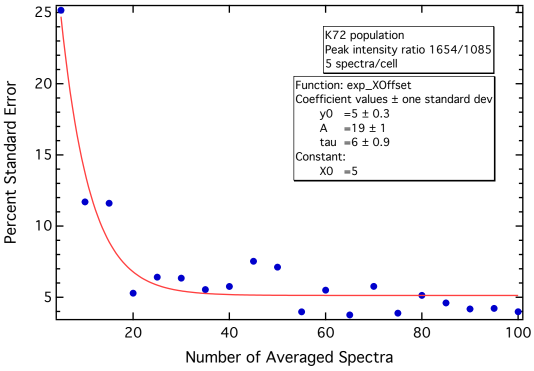

(c) K72–primary

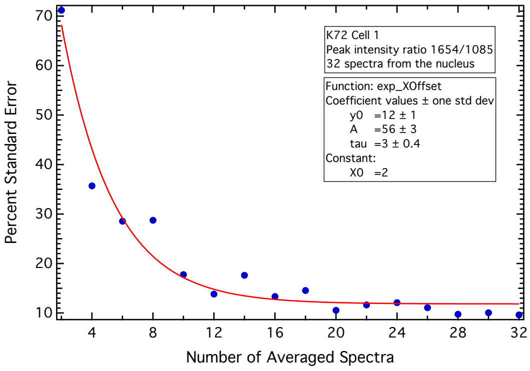

(d) K72 cell one

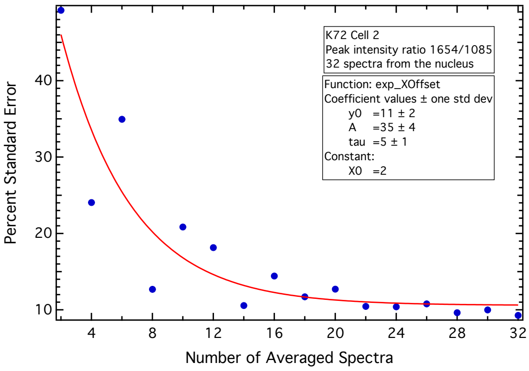

(e) K72 cell two

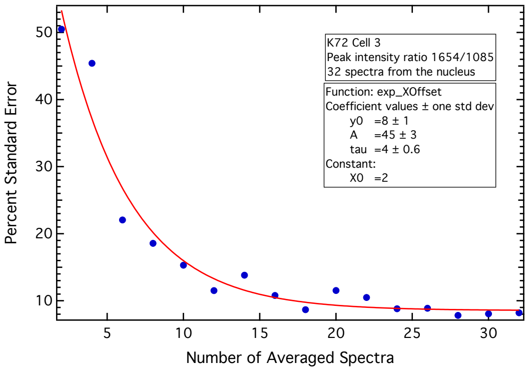

(f) K72 cell three

FIG. S20. 1654/1085 PIR percentage standard error (%SE) convergence results as a function of the number of increasing spectra in the spectral average for the HDF, CD317+MSC and K72-primary cell-line populations [Figs. (a), (b) & (c), respectively], and for the K72 cells 1, 2 and 3 [Figs. (d), (e) & (f), respectively]. A decaying exponential function $[y = y_0 + A \exp\frac{x-x_0}{\tau}]$ was used to fit the data. Each sub-figure shows the fitted values for the function parameters, where tau $= \tau$ denotes the decay constant.

G. Substrates

CaF_2 slides were preferred to glass slides as glass creates a large background against the weak-signal Raman bands from the cells (Fig. S21). In comparison, the single crystal CaF_2 sample has a single peak observed at $\sim$320 cm^{-1}, which is outside of the acquisition range for the cell data, thereby ensuring that the cell signature is above the otherwise low background.

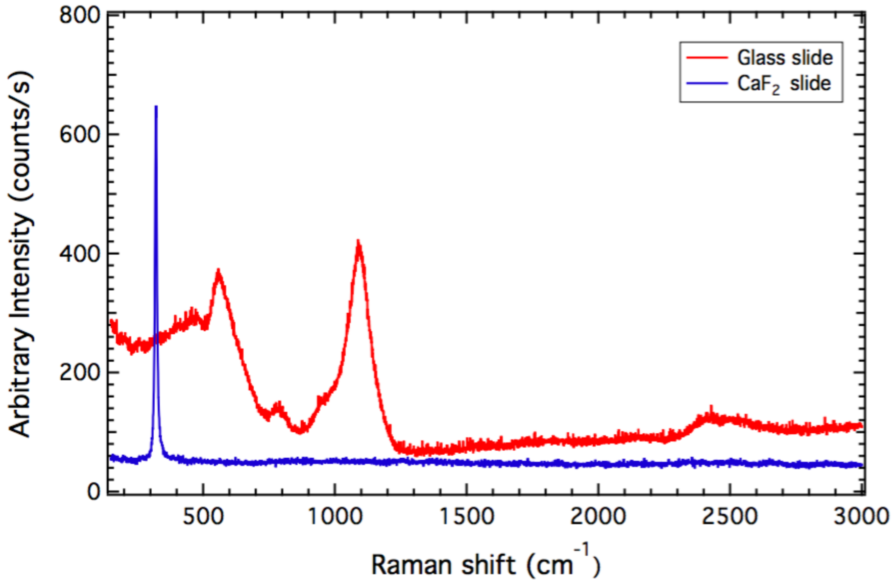

FIG. S21. Comparison of Raman spectra from a glass microscope slide versus that obtained from a CaF_2 microscope slide. The acquisition settings were the same as those used to obtain the cell spectra in this study.

H. Normalised vs. non-normalised spectra and PIR comparisons

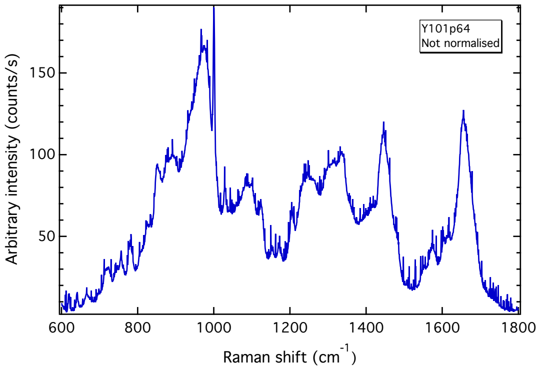

(a)

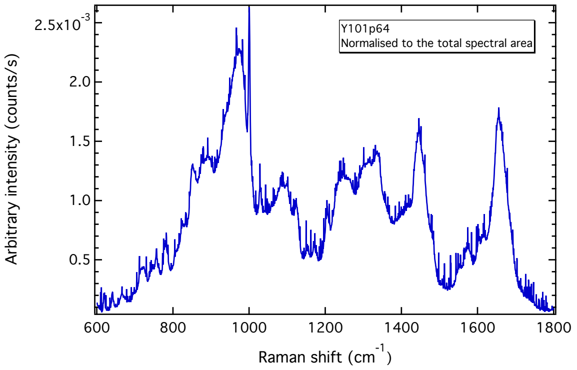

(b)

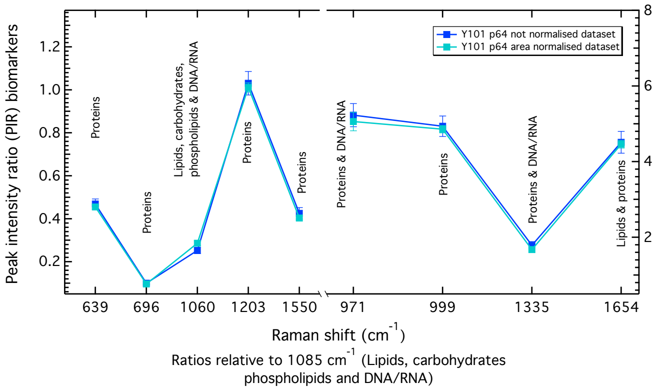

(c)

FIG. S22. (a) Non-normalised composite spectra comprising the spectral average, (b) normalised composite spectra comprising the spectral average, and (c) the derived PIRs obtained for the Y101 p64 population, where a statistically converged amount of constituent spectra (100) have been used. The comparisons demonstrate the robustness of the statistical representation of the experiment.

Supplementary Information References

[S1] James, S., Fox, J., Afsar,i F., Lee, J., Clough, S., Knight, C., Ashmore, J., Ashton, P., Preham, O., Hoogduijn. M., Ponzoni, R. A. R., Hancock, Y., Coles, M. & Genever, P. Multiparameter analysis of human bone marrow stromal cells identifies distinct immunomodulatory and differentiation-competent subtypes. *Stem Cell Reports* **4**, 1004–1015 (2015).

[S2] Movasaghi, Z., Rehman, S. & Rehman, I. U. Raman spectroscopy of biological tissues. *Applied Spectroscopy Reviews* **42**, 493–591 (2007).

[S3] Notingher, I., Bisson, I., Bishop, A. E., Randle, W. L., Polak, J. M. P. & Hench, L. L. In situ spectral monitoring of mRNA translation in embryonic stem cells during differentiation in vitro. *Analytical Chemistry* **76**, 3185–3193 (2004).

[S4] Chan, J. W., Lieu, D. K., Huser, T. & Li, R. A. Label-free separation of human embryonic stem cells and their cardiac derivatives using Raman spectroscopy. *Analytical Chemistry* **81**, 1324–1331 (2009).

[S5] Schulze, H. G., Konorov, S. O., Piret, J. M., Blades, M. W. & Turner, R. F. B. Label-free imaging of mammalian cell nucleoli by Raman spectroscopy. *Analyst* **138**, 3416–3423 (2013).

[S6] Nijssen, A., Schutt, T. C. B., Heule, F., Caspers, P. J., Hayes, D. P., Neumann, M. H. A. & Puppels, G. J. Discriminating basal cell carcinoma from its surrounding tissue by Raman spectroscopy. *The Journal of Investigative Dermatology* **119**, 64–69 (2002).

[S7] Schulze, H. G., Koronov, S. O., Caron, N. J., Piret, J. M., Blades, M. W. & Turner, R. F. B. Assessing differentiation status of human embryonic stem cells nonivasively using Raman microspectroscopy. *Analytical Chemistry* **82**, 5020–5027 (2010).

[S8] McManus, L. L., Burke, G. A., McCafferty, M. M., O'Hare, P., Modreanu, M., Boyd, A. R. & Meenan, B. J. Raman spectroscopy monitoring of the cell osteogenic differentiation of human mesenchymal stem cells. *Analyst* **136**, 2471–2481 (2011).

[S9] Meurens, M., Wallon, J., Tong, J., Noel, H. & Haot, J. Breast cancer detection by Fourier transformed infrared spectrometry. *Vibrational Spectroscopy* **10**, 341–346 (1996).

[S10] Matthaus, C., Bird, B., Miljkovic, M., Chernenko, T., Romeo, M. & Diem, M. in Methods in Cell Biology—Biophysical Tools for Biologists, Vol. 89, *Infrared and Raman Microscopy in Cell B* 275–308 (Academic Press Elsevier, 2008).

[S11] Mitchell, A., Ashton, L., Yang, X.B., Goodacre, R., Tomlinson, M. J., Smith, A. & Kirkham, J. Aseptic Raman spectroscopy can detect changes associated with the culture of human dental pulp stromal cells in osteoinductive culture. *Analyst* **140**, 7347–7354 (2015).

[S12] Hashimoto, A., Chiu, L., Sawada, K., Ikeuchi, T., Fujita, K., Takedachi, M., Yamaguchi, Y., Kawata, S., Murakami, S. & Tamiya, E. In situ Raman imaging of osteoblastic mineralization. *Journal of Raman Spectroscopy* **45**, 157–161 (2014).
